# Sequences, Annotation and Single Nucleotide Polymorphism of the Major Histocompatibility Complex in the Domestic Cat

**DOI:** 10.1371/journal.pone.0002674

**Published:** 2008-07-16

**Authors:** Naoya Yuhki, James C. Mullikin, Thomas Beck, Robert Stephens, Stephen J. O'Brien

**Affiliations:** 1 Laboratory of Genomic Diversity, National Cancer Institute at Frederick, Frederick, Maryland, United States of America; 2 Genome Technology Branch, National Human Genome Research Institute, National Institutes of Health, Bethesda, Maryland, United States of America; 3 SAIC-Frederick, Laboratory of Genomic Diversity, National Cancer Institute at Frederick, Frederick, Maryland, United States of America; 4 SAIC-Frederick, Advanced Biomedical Computing Center, National Cancer Institute at Frederick, Frederick, Maryland, United States of America; University of Uppsala, Sweden

## Abstract

Two sequences of major histocompatibility complex (MHC) regions in the domestic cat, 2.976 and 0.362 Mbps, which were separated by an ancient chromosome break (55–80 MYA) and followed by a chromosomal inversion were annotated in detail. Gene annotation of this MHC was completed and identified 183 possible coding regions, 147 human homologues, possible functional genes and 36 pseudo/unidentified genes) by GENSCAN and BLASTN, BLASTP RepeatMasker programs. The first region spans 2.976 Mbp sequence, which encodes six classical class II antigens (three DRA and three DRB antigens) lacking the functional DP, DQ regions, nine antigen processing molecules (DOA/DOB, DMA/DMB, TAPASIN, and LMP2/LMP7,TAP1/TAP2), 52 class III genes, nineteen class I genes/gene fragments (FLAI-A to FLAI-S). Three class I genes (FLAI-H, I-K, I-E) may encode functional classical class I antigens based on deduced amino acid sequence and promoter structure. The second region spans 0.362 Mbp sequence encoding no class I genes and 18 cross-species conserved genes, excluding class I, II and their functionally related/associated genes, namely framework genes, including three olfactory receptor genes. One previously identified feline endogenous retrovirus, a baboon retrovirus derived sequence (ECE1) and two new endogenous retrovirus sequences, similar to brown bat endogenous retrovirus (FERVmlu1, FERVmlu2) were found within a 140 Kbp interval in the middle of class I region. MHC SNPs were examined based on comparisons of this BAC sequence and MHC homozygous 1.9× WGS sequences and found that 11,654 SNPs in 2.84 Mbp (0.00411 SNP per bp), which is 2.4 times higher rate than average heterozygous region in the WGS (0.0017 SNP per bp genome), and slightly higher than the SNP rate observed in human MHC (0.00337 SNP per bp).

## Introduction

The major histocompatibility complex (MHC) is one of the most extensively analyzed regions in the genome due to the fact that this region encodes the most important molecules in immune function, namely class I and class II antigens, and also other important molecules such as chemical sensing genes (olfactory receptor gene complex), its escort gene, and POU5F1 gene involved in iPS stem cells [1]–[4]. Recently, the human MHC, HLA haplotypes were sequenced in the HLA haplotype project [5]–[10]. Eight different HLA – homozygous haplotypes' DNA sequences were determined in order to shed a light on MHC–linked diseases and evolutionary history. These BAC-based sequencings are necessary to examine the details in the regions of the genome, where gene duplications, deletions and selections occurred many times, because the genome project, especially in the human genome, was carried out using a mixture of DNA sources [11]. The same will be true in genome projects in other outbred species. The domestic cat serves excellent animal models to study at least three RNA viruses in humans. Feline leukemia virus (FeLV) shares similarly to human leukemia viruses (HTLV I & II) [12]. Feline immunodeficiency virus is considered to cause similar symptoms to human AIDS in a natural host, the domestic cat [13]–[16]. Feline infectious peritonitis virus belongs to the same virus group (corona virus) as human SARS virus [17]. To study host-defense mechanisms, in this animal model, we previously analyzed and reported (i) approximately 750 kbp class II region in feline MHC (FLA) [18], (ii) the unique FLA structure with a single chromosomal split at the TRIM gene family region, and chromosome inversion [19], and (iii) comparison of three MHCs, HLA, DLA, and FLA using human sequence, canine MHC homozygous genomic sequence and feline 3.3 Mbp draft sequence based on BAC shotgun sequences [20]. In this manuscript, much detail of FLA gene contents, promoter structures of predicted functional class I and class II genes, proportional scale comparisons of four mammalian MHCs (domestic cat, human, mouse, dog) and one marsupial MHC (opossum) are presented. SNPs (single nucleotide polymorphisms) between the MHC homozygous sequence of the lightly covered (1.9×) domestic cat genome shotgun sequence and this BAC-based MHC sequence were also analyzed to compare the degree and mode of the MHC divergence. In addition, two haplotype BAC-based sequences in functional class II DR region in the domestic cat were analyzed.

## Materials and Methods

### BAC sequencing and assembly

BAC clones from RPCI86 domestic cat BAC library [21] were selected based on FLA BAC map previously described [21]. Shotgun libraries were made using the sonication method [18]. Sequencing reactions were made from both ends of the plasmid vector using BigDye v1.0 chemistry (ABI). Electrogram files (ab1 files) were ftp-transferred to an ABCC ncisgi high speed computer, analyzed by Phred base caller, assembled by Phrap assembler and finished sequence assembly by Consed13 autofinish programs [22]–[25]. The final assembly of these BAC sequence contigs were made using Crossmatch program (http://www.phrap.org/phredphrapconsed.html). The following BAC clones were analyzed for class III and proximal, central class I FLA regions in fcaB2qcen; 181p11, 116b21, 539f24, 162h14, 207i7, 20f19, 18a04, 141b1, 97q9, 410h15, 261j7, 469m20, 515g14, 167d5, 117c16, 27j10, 194g24, 253j16, 292m22, 455a7, 454a5, 148o13, 117e16, 329i22. The following BAC clones were analyzed for class I distal region in fcaB2pter with the order from a telomere of fcaB2 short arm, 46j10, 596j24, 269n17, 221p5. More than sequence quality value 20 was used for the final assembly. The first assembly from class III through central class I regions was connected with previously published [18] class II region sequence (758 Kbp) using Crossmatch program.

### Gene annotation

Sequences were first masked by Repeatmasker program. Gene annotation was made using GENSCAN [26] coding prediction plus BLASTP and BLASTN programs [27], also using megablast for the entire sequences against the latest human Refseq database. Class I, MIC, BAT1, olfactory receptor, MOG, TRIM26, 15, 10 gene annotation was made using human transcripts or FLA class I mRNA sequence (FLAI-A24) [28] by bl2seq [29] and results were parsed using Perl scripts. Repeat sequences were analyzed using Repeatmasker and STR finder programs. These data was graphically presented using Advanced PIPmaker program [30].

### Dotplot analysis

Blastz program [31] was used to generate raw blastz output with parameters: Y = 3400, H = 2200, W = 8, B = 2, K = 3000, C = 0, M = 83886080, P = 0 and this output and two sequences were submitted to Advanced PIPmaker website (http://pipmaker.bx.psu.edu/cgi-bin/pipmakeradvanced).

### SNP analysis

The 1.9× feline WGS contigs [32] were aligned with BAC MHC sequence using CROSSMATCH program and SNP was found between sequences selected by reciprocal best matches (>90% sequence identity) and with more than Quality value = 15 [33].

### DR haplotypes

BAC clones of 152N13–244j14–16i4 from B2qCen side were sequenced by the shotgun method described above and analyzed by the methods of GENSCAN, Spidey [34] and Genwise [35] for annotation. A sequence assembled from this DR haplotype 2 region was compared with a sequence from DR haplotype 1 region previously published [18].

### Comparisons of MHC structures

Sequences of MHC from four species: human, mouse, dog, and opossum which span from KIFC1 gene through UBD plus three olfactory receptor genes were extracted from UCSC Genome Browser. Gene coordinates tables from UCSC site were parsed by Perl script and gene organizations were graphically plotted by R script (http://cran.R-project.org).

### Transcription factor binding sites in promoter regions of predicted functional feline class I and class II DR genes

Sequences totaling 6 kb (5 kb upstream and 1 kb downstream) from a potential translation start site (ATG) of predicted functional feline class I genes (FLAI-E, I-H, I-K) and class II DR genes (FLA-DRA1, DRA2, DRA3, DRB1, DRB3, DRB4) in addition to human HLA-A, -B, -C class I genes and HLA-DRA, DRB1, DRB3 class II genes were analyzed for the presence of potential transcription factor binding sites using Match TM program with TRANSFAC 7.0 database (http://www.gene-regulation.com/pub/databases.html). In addition, S-Y-module sequences of human HLA class II and I genes were used to screen above 6 kb sequences with b12 seq [29] with parameters, MATCH = 1, MISMATCH = −1, GAP OPEN 5, GAP EXTENSION 2, X_DROP OFF 0, EXPECT 10.00, WORDSIZE 7.

## Results

### Sequence

2,975,516 bp and 381,545 bp sequences were assembled for two FLA regions on the pericentromeric and subtelomeric positions of feline chromosome FcaB2. The first sequence covers from KIFC1 gene in the extended class II region through the entire class II, class III and a part of class I regions from the point adjacent to BAT1 gene through HLA-B, -C class I corresponding region, TRIM39 plus HLA-92 (HLA-L) region to alpha satellite-rich region. The second sequence covers from telomeric repeats rich region through TRIM 26/15/10 genes to the third olfactory receptor like gene (GenBank accession Nos. EU153401, EU153402).

### Annotation

**Figure 1:**
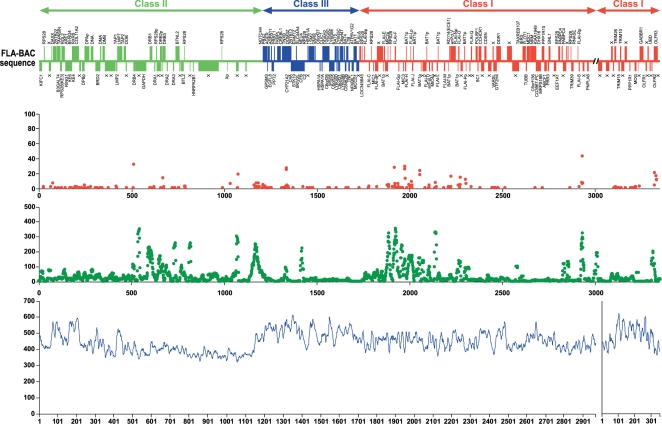
Gene Organization, SNP level, GC contents in FLA. (A) Gene organization of FLA. Genes with forward orientation, which towards to telomere in human HLA, but towards to centromere in FLA, and away from telomere in distal class I region in FLA were placed above the solid line. A position of the ancient chromosome break and an inversion was indicated by double slashed lines and genes with opposite orientation were placed below the solid line. (B) Coding (CDS) SNPs. CDS SNPs were counted based on exon structure of each gene. Pseudogenes CDS SNPs were omitted. No. of SNPs per 10 kbp were plotted. (C) Single nucleotide polymorphism (SNP). SNP was counted in 10 Kbp window and shift 1 Kb. (D) GC content. GC content was counted in 10 Kbp window and shift 1 Kb and number was plotted.

**Figure 2:**
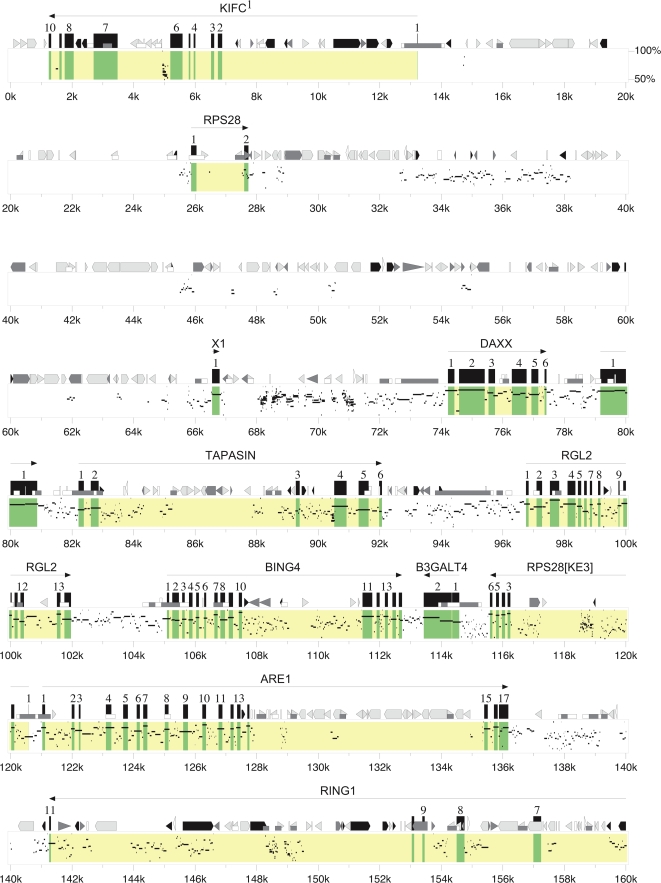
Percent Identity Plots between FLA and HLA. (A) Percent identity Plot of FLA extend class II, classical class II, class III, proximal and central class I regions. Genes and exons were highlighted with yellow and green colors, respectively. Gene, exon, UTR, simple repeats, MIR, other SINE, LINE1, LINE2, LTR, other repeats, CpG/GpC ratios were indicated. FLA sequence was compared with human HLA 6COX sequence. (B) Percent Identity Plot of FLA distal class I region. Same methods and criterions were used as in Figure 2(A). Only Figure 2A-1 was included in this text. The rest of Figure 2A and 2B can be found in Supporting Information File, Figure 2AB_All.tar.bzip2.

The entire gene coordinates, and possible functions are listed in Table 1. Gene organization and GC level was depicted in Figure 1. Detailed graphic presentation for exon-intron structure, orientation, repeat sequence, CpG island and sequence identity level to human HLA-6 COX 4.72 Mbp sequence was organized in Figures 2. (Figure 2A-1 was shown in the main text. Please see Figure S1 supporting file).

**Table 1:** Predicted Genes, their Functions and Coordinates in *FLA*.

Gene	Functional/physiological properties/other name/structure	Orientation	Start	End	Length
*KIFC1*	kinesin family member C1	−	1243	13226	11983
*RPS28*	ribomal protein S28	+	25869	27731	1862
*X1*	unknown: cfa chr12.6 - 029.a N-SCAN gene prediction	+	66551	66793	242
*DAXX*	death-associated protein 6	+	74219	77417	3198
*ZBTB22*	zinc finger and BTB domain containing 22	+	79170	80865	1695
*TAPBP*	TAP binding protein (tapasin)	+	82211	92070	9859
*RGL2*	ral guanine nucleotide dissociation stimulator-like 2	+	96755	101963	5208
*BING4*	WD domain	+	105084	112722	7638
*B3GALT4*	UDP-Gal:betaGlcNAc beta 1,3-galactosyltransferase, polypeptide 4	−	113431	114583	1152
*RPS28[KE3]*	ribomal protein S28	−	115583	120594	5011
*ARE1*	Yeast sac2 homolog, suppressor of actin mutant 2, Sacm2l, coiled coil structure	+	121032	136177	15145
*RING1*	ring finger protein 1	−	141255	167593	26338
*KE6*	Steroid dehydrogenase-like protein (estradiol 17 beta-dehydrogenase 8)	−	170695	172885	2190
*KE4*	Transmembrane protein with histidine-rich charge clusters	−	173342	175211	1869
*RXRB*	retinoid X receptor, beta	+	177083	181628	4545
*COL11A2*	collagen, type XI, alpha 2	+	184143	212170	28027
*X2*	unknown: cfa chr12: 5,766,607–5,773,167	−	216881	223065	6184
*DPBp*	Class II antigen beta chain, pseudogene	−	231611	242652	11041
*DPAp*	Class II antigen alpha chain, pseudogene	+	245150	268064	22914
*DNA*	DOA, heterodimerize with DOB in pre-B cells, peptide loading for class II antigen at low PH	+	278617	289676	11059
*BRD2*	bromodomain containing 2	−	303996	327744	23748
*DMA*	Nonclassical class II antigen, alpha chain, peptide loading for class II antigen	+	329474	332504	3030
*DMB*	Nonclassical class II antigen, beta chain, heterodimer with DMA,peptide loading for class II antigen	+	342380	351767	9387
*LMP2*	Proteosome subunit to cleave peptides for class I antigen	−	416025	419082	3057
*TAP1*	transporter 1, ATP-binding cassette, sub-family B (MDR/TAP)	+	421610	428032	6422
*LMP7*	Proteosome subunit to cleave peptides for class I antigen	+	431491	433586	2095
*TAP2*	transporter 2, ATP-binding cassette, sub-family B (MDR/TAP)	+	435278	446029	10751
*DOB*	Nonclassical class II antigen, beta chain, heterodimer with DNA, H2-IAB2 in mouse	+	456252	465656	9404
*DRB4*	Class II antigen, beta chain	−	509012	514820	5808
*GAPDH*	Glycerol aldehyde phosphodehydrase, pseudogene	−	563173	575620	12447
*DRB1*	Class II antigen, beta chain	+	596698	607755	11057
*DRA1*	Class II antigen, alpha chain	−	621375	624059	2684
*RPS28p*	ribomal protein S28 gene fragment	+	631565	633427	1862
*DRB2p*	Class II antigen, beta chain pseudogene with intron 1 and exon 2	+	652000	659950	7950
*DRB3*	Class II antigen, beta chain	+	664799	679467	14668
*DRA2*	Class II antigen, alpha chain	−	688714	691459	2745
*DRA3*	Class II antigen, alpha chain	−	722112	725240	3128
*BTL2*	butyrophilin-like 2 (MHC class II associated)	+	735556	754731	19175
*BTL2*	butyrophilin-like 2 (MHC class II associated)	−	765965	777458	11493
*RPS28*	ribomal protein S28	+	782864	783037	173
*HNRPA2B1*	heterogeneous nuclear ribonucleoprotein A2/B1	−	829773	830860	1087
*RPS28*	ribomal protein S28	+	984596	984769	173
*NOTCH4*	Notch homolog 4 (Drosophila)	+	1206316	1229484	23168
*GPSM3*	G-protein signalling modulator 3 (AGS3-like, C. elegans)	−	1230443	1231562	1119
*PBX2*	pre-B-cell leukemia transcription factor 2	+	1234538	1236556	2018
*AGER*	advanced glycosylation end product-specific receptor	+	1238751	1241285	2534
*AGPAT1*	1-acylglycerol-3-phosphate O-acyltransferase 1 (lysophosphatidic acid acyltransferase, alpha)	+	1250819	1252976	2157
*EGFL8*	EGF-like-domain, multiple 8	−	1254290	1255788	1498
*PPT2*	palmitoyl-protein thioesterase 2	−	1258652	1263918	5266
*PRRT1*	proline-rich transmembrane protein 1	+	1267796	1269170	1374
*FKBPL*	FK506 binding protein like	+	1284306	1285355	1049
*CREBL1*	cAMP responsive element binding protein-like 1	+	1285915	1300603	14688
*TNXB*	tenascin XB	+	1310433	1357909	47476
*CYP21A2*	cytochrome P450, family 21, subfamily A, polypeptide 2	−	1358128	1360631	2503
*C4A*	complement component 4A (Rodgers blood group)	−	1363648	1378177	14529
*STK19*	serine/threonine kinase 19	−	1379597	1384827	5230
*DOM3Z*	dom-3 homolog Z (C. elegans)	+	1385598	1387305	1707
*SKIV2L*	superkiller viralicidic activity 2-like (S. cerevisiae)	−	1387465	1394012	6547
*CFB*	complement factor B	−	1394591	1402014	7423
*C2*	complement component 2	−	1402258	1415710	13452
*ZBTB12*	zinc finger and BTB domain containing 12	+	1448172	1449551	1379
*EHMT2*	euchromatic histone-lysine N-methyltransferase 2	+	1454322	1466627	12305
*SLC44A4*	solute carrier family 44, member 4	+	1467409	1479530	12121
*NEU4*	Neuraminidase 4	+	1480105	1483273	3168
*RPS28*	ribomal protein S28	+	1488926	1489106	180
*HSPA1A*	heat shock 70 kDa protein 1A	−	1513315	1515240	1925
*HSPA1A*	heat shock 70 kDa protein 1A	−	1524090	1526015	1925
*HSPA1A*	heat shock 70 kDa protein 1A	+	1528138	1529946	1808
*LSM2*	LSM2 homolog, U6 small nuclear RNA associated (S. cerevisiae)	+	1532633	1537716	5083
*VARS*	valyl-tRNA synthetase	+	1539067	1553500	14433
*C6orf27*	chromosome 6 open reading frame 27	+	1555291	1563506	8215
*C6orf26*	chromosome 6 open reading frame 26	−	1564291	1565750	1459
*MSH6*	mutS homolog 6 (E. coli)	−	1566395	1590460	24065
*CLIC1*	chloride intracellular channel 1	+	1594157	1598924	4767
*DDAH2*	dimethylarginine dimethylaminohydrolase 2	+	1600599	1602528	1929
*C6orf25*	chromosome 6 open reading frame 25	−	1604591	1606252	1661
*LY6G6C*	lymphocyte antigen 6 complex, locus G6C	+	1607799	1610137	2338
*LY6G6D*	lymphocyte antigen 6 complex, locus G6D	−	1611498	1613549	2051
*LY6G6E*	lymphocyte antigen 6 complex, locus G6E	+	1615087	1616230	1143
*C6orf21*	chromosome 6 open reading frame 21	−	1618100	1620941	2841
*BAT5*	HLA-B associated transcript 5	+	1623128	1636811	13683
*LY6G5C*	lymphocyte antigen 6 complex, locus G5C	+	1642789	1645897	3108
*LY6G5B*	lymphocyte antigen 6 complex, locus G5B	−	1649307	1650335	1028
*CSNK2B*	casein kinase 2, beta polypeptide	−	1651325	1654509	3184
*BAT4*	HLA-B associated transcript 4	+	1656917	1658511	1594
*C6orf47*	chromosome 6 open reading frame 47	+	1660571	1661426	855
*LTB*	lymphotoxin beta (TNF superfamily, member 3)	+	1663590	1665305	1715
*TNF*	tumor necrosis factor (TNF superfamily, member 2)	−	1668300	1670066	1766
*LTA*	lymphotoxin alpha (TNF superfamily, member 1)	−	1672461	1673412	951
*NFKBIL1*	nuclear factor of kappa light polypeptide gene enhancer in B-cells inhibitor-like 1	−	1685899	1694249	8350
*ATP6V1G2*	ATPase, H+ transporting, lysosomal 13 kDa, V1 subunit G2	+	1695942	1697113	1171
*BAT1*	HLA-B associated transcript 1	+	1699863	1710709	10846
*MCCD1*	mitochondrial coiled-coil domain 1	−	1711244	1714362	3118
*X3*	unknown: cfa chr12: 4,026,686–4,028,708	−	1716465	1722099	5634
*LOC345645*	similar to peptidase (prosome, macropain) 26S subunit, ATPase 1	−	1722382	1723468	1086
*FLAI-A*	non classical class I molecule	+	1727339	1733904	6565
*RPS28*	ribomal protein S28	+	1741091	1741264	173
*FLAI-Bp*	Classical class I antigen gene fragment	+	1754279	1755539	1260
*FLAI-C*	non classical class I molecule	−	1778815	1781240	2425
*RPS28*	ribomal protein S28	+	1791524	1791697	173
*MIC1*	MHC class I releated gene 1	−	1802050	1803673	1623
*FLAI-Dp*	Classical class I antigen gene fragment	−	1812028	1826561	14533
*X4*	unknown	−	1816059	1819441	3382
*X5*	unknown: cfa chr12: 3,304,294–3,451,219	+	1822307	1852375	30068
*BAT1p*	BAT1 fragement	−	1854809	1855450	641
*FLAI-E*	Classical class I antigen ( with long cytoplasmic tail)	+	1859534	1862925	3391
*X6*	unknown	−	1865368	1878940	13572
*MIC2p*	MHC class I releated gene 2 fragment	+	1880418	1880645	227
*RPS28*	ribomal protein S28	+	1892390	1892515	125
*FLAI-F*	non classical class I molecule	+	1918438	1921808	3370
*FLAI-Gp*	Classical class I antigen gene fragment	−	1938101	1940148	2047
*MIC3*	MHC class I releated gene 3	−	1962637	1964257	1620
*FLAI-H*	Classical class I antigen	−	1973577	1976991	3414
*BAT1p*	BAT1 fragement	+	1979546	1980192	646
*FLAI-Ip*	Classical class I antigen gene fragment	+	2003006	2003225	219
*FLAI-J*	non classical class I molecule	−	2011852	2015260	3408
*BAT1p*	BAT1 fragement	+	2017763	2018402	639
*BAT1p*	BAT1 fragement	−	2059127	2059750	623
*FLAI-K*	Classical class I antigen	−	2083736	2087122	3386
*BAT1p*	BAT1 fragement	+	2089903	2090242	339
*FERVmlu2*	endogenous retrovirus similar to brown bat (Motis Lucifugus) endogenous retrovirus 2	−	2103256	2105890	2634
*MIC4p*	MHC class I releated gene 4 fragment	−	2117309	2117536	227
*X7*	unknown: cfa chr10: 6,669,201–6,794,639	−	2117453	2120737	3284
*FLAI-L*	non classical class I molecule	−	2145008	2148428	3420
*BAT1p*	BAT1 fragement	+	2150915	2151554	639
*FLAI-M*	non classical class I molecule	−	2183520	2187094	3574
*BAT1p*	BAT1 fragement	−	2205884	2206254	370
*RD114(ECE1)*	baboon retrovius related endogenous retrovirus	+	2212532	2215463	2931
*FERVmlu1*	endogenous retrovirus similar to brown bat (Motis Lucifugus) endogenous retrovirus 1	+	2219742	2244701	24959
*FLAI-Np*	Classical class I antigen gene fragment	+	2221196	2221264	68
*BAT1p*	BAT1 fragement	−	2258148	2258782	634
*FLAI-O*	non classical class I molecule	+	2260882	2264315	3433
*BAT1p*	BAT1 fragement	+	2293369	2294534	1165
*FLAI-Pp*	Classical class I antigen gene fragment	−	2301870	2302131	261
*FLAI-Q*	non classical class I molecule	+	2329070	2332537	3467
*POU5F1*	POU domain, class 5, transcription factor 1, OCT3	+	2354289	2362844	8555
*SC1*	TCF19, transcription factor 19	−	2364737	2367161	2424
*CCHCR1*	coiled-coil alpha-helical rod protein 1	+	2370568	2382864	12296
*CDSN*	corneodesmosin	+	2401577	2410158	8581
*X8*	unknown: chr12: 3,698,941–3,701,819	+	2446230	2449015	2785
*VARSL*	valyl-tRNA synthetase like	−	2449807	2461727	11920
*GTF2H4*	general transcription factor IIH, polypeptide 4, 52 kDa	−	2461975	2465896	3921
*DDR1*	discoidin domain receptor family, member 1	+	2466245	2488040	21795
*TAXREB107*	TAX response lement-binding protein	+	2527942	2547435	19493
*IER3*	immediate early response 3	+	2607659	2607996	337
*FLOT1*	flotillin 1	+	2610071	2622235	12164
*TUBB*	tubulin, beta	−	2625114	2628405	3291
*MDC1*	mediator of DNA damage checkpoint 1	+	2630309	2644393	14084
*NRM*	nurim (nuclear envelope membrane protein)	+	2648569	2653735	5166
*KIAA1949*	KIAA1949	+	2653850	2660634	6784
*DHX16*	DEAH (Asp-Glu-Ala-His) box polypeptide 16	+	2663783	2678921	15138
*C6orf136*	chromosome 6 open reading frame 136	−	2679243	2683432	4189
*CG3967-PC*	Drosophila melanogaster protein Cg3967-pc homolog	−	2683765	2697339	13574
*MRPS18B*	mitochondrial ribosomal protein S18B	−	2698088	2703714	5626
*PPP1R10*	protein phosphatase 1, regulatory subunit 10	+	2711308	2722165	10857
*ABCF1*	ATP-binding cassette, sub-family F (GCN20), member 1	−	2724683	2735486	10803
*PRR3*	proline rich 3	−	2743092	2747113	4021
*GNL1*	guanine nucleotide binding protein-like 1	+	2748233	2755021	6788
*EEF1A1*	eukaryotic translation elongation factor 1 alpha 1	−	2779084	2786223	7139
*RPS28*	ribomal protein S28	+	2800184	2807333	7149
*PABPC4*	poly(A) binding protein, cytoplasmic 4 (inducible form)	+	2818343	2818789	446
*PABPC4*	poly(A) binding protein, cytoplasmic 4 (inducible form)	+	2820533	2821355	822
*X9*	unknown: PLEC1, PLECTIN1	−	2825427	2840447	15020
*X10*	unknown: SLC12AL Intron, sodium potassium chloride cotransporter2	+	2842351	2843559	1208
*RPS28*	ribomal protein S28	+	2850617	2852479	1862
*RNASE*	Ribonuclease	+	2867225	2867494	269
*TRIM39*	tripartite motif-containing 39	−	2870168	2879926	9758
*FLAI-Rp*	Classical class I antigen gene fragment	+	2905161	2905383	222
*FLAI-S*	non classical class I molecule	−	2910524	2913237	2713
*PNPLA6*	Patatin-like phospholipase domain containing 6	−	2956238	2961356	5118
PeriCentromic Region and chromosomal break and inversion					
Subtelomeric Region					
X11	unknown: cfa chr6: 27,705,326–27,717,672	−	7700	29900	22200
TRIM26	tripartite motif-containing 26 protein	+	102400	111040	8640
TRIM15	tripartite motif-containing 26 protein	−	125740	132100	6360
TRIM10	tripartite motif-containing 26 protein	+	134820	140560	5740
X12	unknown: cfa chr35: 29,351,535–29,362,511	−	142140	158060	15920
PPP1R11	protein phosphatase 1, regulatory (inhibitor) subunit 11	−	189360	193120	3760
MOG	myelin oligodendrocyte glycoprotein	−	219300	228580	9280
GABBR1	gamma-aminobutyric acid (GABA) B receptor 1	+	248040	268920	20880
OLFR1	Olfactory receptor	−	282860	289000	6140
UBD	ubiquitin	+	299600	301440	1840
X13	unknown	−	303120	303740	620
OLFR2	Olfactory receptor	−	316640	317400	760
OLFR3	Olfactory receptor	+	330520	352180	21660

#### Extended and classical class II region

Extended class II region spans 230 Kbp from KIFC1 gene to the point adjacent to DPB pseudogene. Fourteen human gene homologues and 2 unknown coding regions were found. Classical class II region spans 884 Kbp. Twenty-five human gene homologues were found in the region defined from DPB pseudogene to the point adjacent region of NOTCH4 gene. Annotation and sequence of a part of this region, (KIFC1, previously assigned as HSET to BTNL2), were described elsewhere [18]. Briefly, a pair of DPA, B pseudogenes, 3 pairs of DRA, B genes were identified with one DRB pseudogene. A set of genes which are involved in antigen processing, including a pair of DOA, DOB, DMA, and B genes, two antigen transporter genes, TAP1, 2, and protease genes, LMP2, 7 were found. In addition, two butylophillin genes, BTNL2, BTL2, and BRD2 (previously assigned as RING3) genes were found.

#### Class III region

FLA class III region spans 520 Kbp which encodes fifty-one human gene homologues and two unknown coding regions.

#### Class I region

FLA class I region were classified as three subregions based on chromosomal localization and gene contents. The first class I region, proximal class I region spans 600 Kbp from the first class I gene (FLAI-A) to the last class I gene (FLAI-Q) in this HLA-B, -C corresponding region adjacent to the class III region. This region encodes seventeen class I genes/gene fragments based on sequence alignments with full length feline class I cDNA sequence. Eight BAT1 gene fragments are located in the vicinity of class I genes. Three RPS28 gene fragments, four class I-related (MIC) genes or gene fragments and four unknown coding regions were also identified.

The second class I subregion, a central class I region, spans 600 kb region from POU5F1 (previously assigned as OCT3) gene to the alpha satellite repeat-rich pericentromeric region. There are 32 human gene homologues including two class I gene/gene fragments in HLA-92 (HLA-L) region, three unknown coding regions. The third class I subregion, distal class I region, spans 360 Kbp from 47 telomere repeats of (TTAGGG) through the third olfactory receptor like gene. This region encodes ten human gene homologues and three unknown coding regions. Three TRIM genes, TRIM26, TRIM15, TRIM10 were identified, however, TRIM40, 31 gene homologues were not recognized. PPPR11 and MOG genes are located in 26 Kbp interval, while in human HLA, these two genes are located with 340 kb interval due to the existence of eleven class I genes/gene fragments as HLA-A region.

#### GC contents

GC contents nearly reached at 60% level in the extended class II, class III, and the distal class I regions. The lowest GC content of nearly 40% was found in the classical class II region and sharply increased at the border of class II and class III regions. The proximal/central class I regions kept GC content at 50% level (Figure 1).

### Repeats

#### Interspersed repeats

Interspersed repeats occupied about thirty-four percentages of MHC region, which is approximately the same level as found in the cat genome (32%), but significantly fewer than human HLA region (48%) or human genome (46%). Table 2 summarized the repeat components in each FLA (sub) class. Though SINE repeat contents are relatively equal in each region ranging from 8 to 14%, the LINE repeat contents are significantly different. The highest LINE contents were found in classical class II and proximal class I regions, (more than 60% of total sequences), where major functional MHC gene amplifications have occurred. The lowest LINE contents were observed in the gene-rich extended class II and class III regions, at approximately 20% level. An intermediate level of LINE contents was found in central and distal class I regions at approximately 40% level.

**Table 2:** Interspersed Elements in *FLA* subregions.

	extended class II	classical class II	class III	proximal class I	central class I	distal class I	*FLA*	cat genome	*HLA*
SINES:	14.86	10.19	11.16	8.04	10.93	8.82	8.53	11.2	17.59
MIRs	2.29	1.69	2.46	0.44	2.07	1.56	1.05	3.10	16.06
LINES:	12.57	34.82	10.84	32.07	23.41	21.02	21.31	14.26	16.59
LINE 1	8.61	32.88	6.86	29.13	19.76	19.39	18.63	10.79	13.35
LINE 2	3.87	1.79	3.58	2.88	3.18	1.44	2.54	2.82	3.09
L3/CR1	0.07	0.14	0.40	0.05	0.36	0.13	0.14	0.36	0.16
LTR elements:	2.84	4.49	1.39	5.17	4.51	4.93	2.69	4.44	10.55
MaLRs	1.90	0.79	0.82	1.51	1.57	0.91	1.04	2.14	2.61
ERVL	0.34	0.88	0.27	1.79	0.57	1.59	0.81	1.21	2.11
ERV classI	0.60	2.74	0.29	1.87	2.32	2.43	0.81	1.05	4.25
DNA elements:	5.23	1.56	1.63	2.56	1.72	1.43	1.62	2.19	2.64
MER1_type	2.30	1.16	0.97	1.73	1.07	0.81	1.31	1.26	1.52
MER2_type	1.46	0.17	0.26	0.83	0.33	0.23	0.14	0.39	0.88
Total of Interspersed	35.48	51.07	25.07	47.84	40.57	36.19	34.14	32.1	48.14

Percentage of sequence (%) was shown in each subregion.

#### Simple Tandem Repeats (STRs)

Frequency of STRs was calculated in each FLA subregion and was compared with results obtained from human HLA 6COX haplotype sequence. These results were summarized in Table 3. A total of 541 STRs (di-, tri-, tetra-, penta-) with more than 12 and 5 perfect repeats for di- and others, e.g. (CA)_12_ and (GGA)_5_, respectively were found in FLA. The frequency of STRs (1 every 6.17 kb) was 50% higher than that in human HLA (1 every 9.93 kb) due to at least 3 times higher frequency of dinucleotide repeats. This trend was more obviously observed in the classical class II region. Approximately 4 times more occurrence of dinucleotide STRs was found in this FLA subregion.

**Table 3:** Simple Tandem Repeats (STRs) in FLA.

*FLA*														
	Extended class II		Classical class II		Class III		Proximal class I		Central class I		Distal class I		Total	
DI	25^a^	9.26^b^	118	8.26	30	17.36	47	13.34	52	11.95	26	13.91	298	11.20
TRI	11	21.05	19	51.30	13	40.07	10	62.70	13	47.79	10	36.15	76	43.91
TETRA	9	25.73	37	26.34	13	40.07	39	16.08	23	27.01	17	21.27	138	24.18
PENTA	2	115.79	7	139.24	8	65.11	7	89.58	4	155.31	1	361.55	29	115.07
TOTAL	47	4.93	181	5.39	64	8.14	103	6.09	92	6.75	54	6.70	541	6.17
size (bp)	231580		974699		520919		627039		621255		361545		3337061	

*HLA* (6 COX)										
	Extended class II		Classical class II		Class III		Class I		Total	
DI	19	12.30	25	35.69	12	54.70	45	46.19	101	38.23
TRI	9	25.96	18	49.57	17	38.61	71	29.28	115	33.57
TETRA	11	21.24	36	24.78	13	50.49	87	23.89	147	26.26
PENTA	5	46.72	3	297.41	3	218.79	15	138.58	26	148.49
TOTAL	44	5.31	82	10.88	45	14.59	218	9.54	389	9.93
size (bp)	233622		892216		656365		2078648		3860851	

a. No. of more than 12 perfect repeats or 5 repeats were counted for dinucleotide (DI) and other STRs (TRI, TETRA, PENTA), respectively.

b. Average interval (kbp) of occurrence of STR was shown.

#### Dotplot analyses of HLA, DLA, FLA

**Figure 3:**
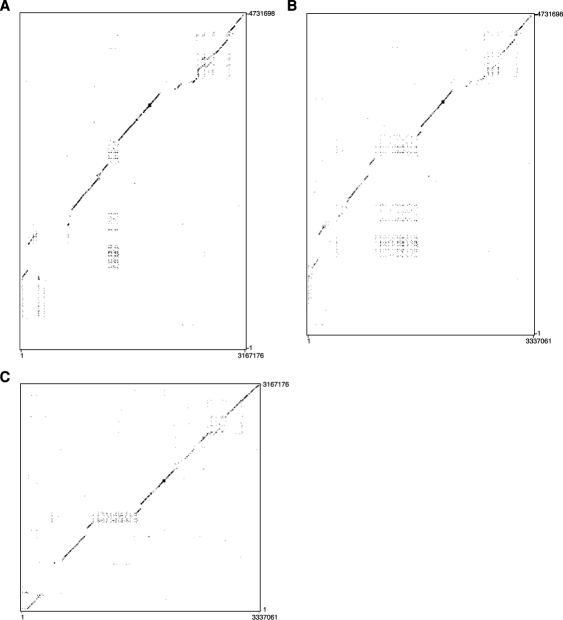
Dotplot analyses. Two FLA sequences were connected based on HLA organization and oriented as follows; Telomeric side of FcaB2q→B2qcen→B2pter→B2p. The centromeric side of two DLA sequences, one on cfa12qcen and the other on cfa35qter were also connected based on HLA organization as follows; telomeric side of cfa12q→cfa12qcen→cfa35qter→cfa35q. (A) Dotplot analysis between DLA (KIFC1 to the third olfactory receptor genes from MOG) and HLA 6COX sequences (X axis vs. Y axis). (B) Dotplot analysis between FLA (KIFC1 to the third olfactory receptor genes from MOG) and HLA 6 COX sequences (X axis vs. Y axis). (C) Dot plot analysis between FLA (X axis) and DLA (Y axis).

MHC sequences spanning from UBD plus three olfactory receptor genes to KIFC1 in HLA, DLA, FLA were compared in pairwise fashion. These analyses, DLA vs. HLA (Figure 3A), FLA vs. HLA (Figure 3B) and FLA vs. DLA (Figure 3C) revealed mosaic structures of highly conserved regions (solid lines), gene duplication (square with dots), deletions (spaces between solid lines in one axis but not in other) and divergent regions (broken lines). Figure 3A and 3B showed similar patterns between DLA vs. HLA and FLA vs. HLA, indicating conserved class II, III, and central class I regions plus class I gene amplifications, though the level of class I gene amplification was lower in DLA due to the fact that only 3 class I genes exit in HLA-B, -C corresponding region. The observation that DLA and FLA lack HLA-A class I region was also evident in this analysis. Figure 3C also showed that FLA and DLA were highly conserved in gene contents and organization except that the level of class I gene amplification was higher in FLA and sequences around pericentromere and subtelomere had highly divergent sequence due to the numerous and different types of gene translocations from other genome sites, resulting in a large broken solid line in these regions.

#### Endogenous retrovirus sequences

**Figure 4:**
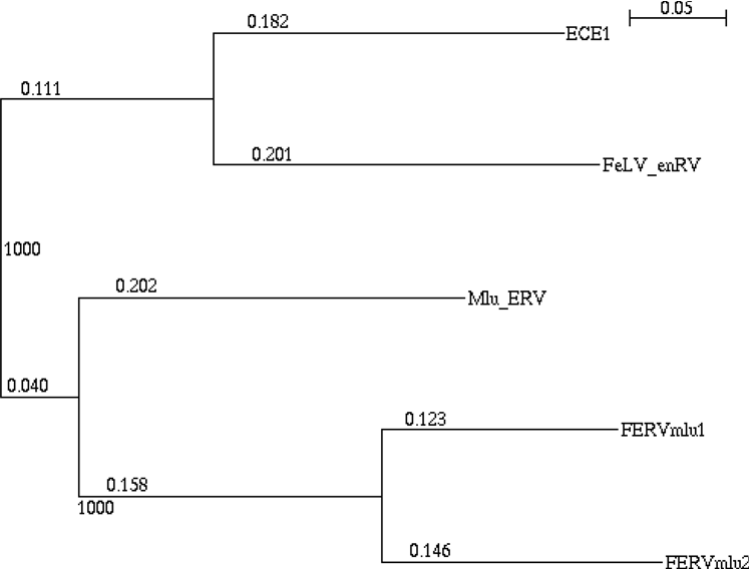
Neighbor-Joining Tree with 1,000 bootstrap for domestic cat endogeneous retrovirus sequences. ECE1 represent RD114 endogeneous retrovirus transmitted from baboon, enFeLV represent a full length FeIV endogeneous retrovirus. enRVMlu represent brown bat retrovirus sequence and FERVmlu1, FERVmlu2 represent new endogeneous genes found in the proximal class I region of FLA in this study.

One of the baboon-derived endogenous retroviruses, ECE1 (RD114) which had 99% sequence identity (1631/1633) with GenBank RD114 (ECE1) AF155060 and two new types of endogenous retroviruses FERVmlu1 and 2, which showed high sequence similarity with recently submitted sequences by an NISC Comparative Sequencing Initiative project of brown bat (Myotis lucifugus) BAC clone (95% sequence identity with 83% coverage, and >85% sequence identity with 83% coverage, respectively) were also recognized within 1401kb region (Figure 4). Detailed open reading frame (ORF) analyses showed FERVmlu1 and 2 have 140 and 19 ORFs which sizes range from 102–900 and 102–516, respectively. The largest ORF of FERVmlu1 encodes 324 amino acid residues which have 70% similarity to a part of recombinant mouse-MuLV/RaLV Pol region, half of retroviral aspartyl protease, DNA binding region and a half of putative active site, however, other ORFs have no significant homology to gag, pol, env regions.

The FERVmlu2 have two Pol-like ORFs. The ORF1 is similar to reverse transcriptase like sequence, in which encodes a DNA binding domain and a putative active site. ORF2 has similarity to an integrase core domain. The third ORF showed a gag – p30 superfamily motif. Nine LTR like sequences were recognized in FERVmlu1 by Repeatmasker program, indicating sequence divergences ranging from 13 to 32% in canine, baboon, chimpanzee endogenous retroviral LTRs.

#### Single nucleotide polymorphism (SNP)

**Figure 5:**
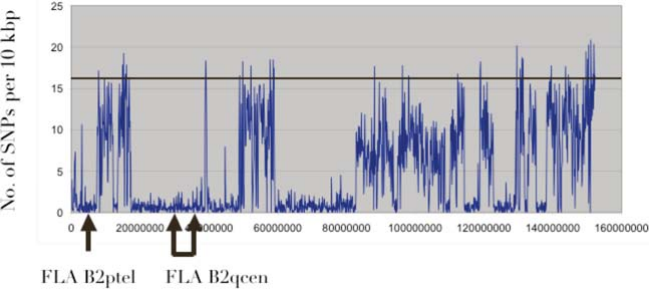
Single Nucleotide Polymorphism (SNP) plot on cat chromosome B2 coordinates. Number of SNPs were counted based on whole genome shotgun sequences and the number of SNPs per 10 Kbp were plotted. A solid line represents average SNP rate (per 10 Kbp) in heterozygous regions of a female Abyssinian cat genome. Areas of FLA were indicated as brackets.

The SNP count plot (Figure 5) in the MHC region from 1.9× cat whole genome shotgun sequence indicated that this region is homozygous. Therefore, MHC BAC sequences were aligned with MHC homozygous 1.9× whole genome shotgun sequence contigs to examine SNP levels in FLA [32]. A total of 2,835,361 bps were aligned with sequence quality value, more than 15 by reciprocal best matches (>90% homology) using the algorithm of Smith-Waterman. This covers more than 85% of the entire FLA region. Distributions of these SNPs and coding SNPs were plotted in Figure 1 and the summary was presented in Table 4. A total of 11,654 SNPs were identified by this method. FLA SNP rate was slightly higher than the rate of two HLA haplotypes (0.00411 vs. 0.00337), and more than 2 times higher than genome wide regions of SNP rate (0.0017) found in regions of heterozygous cat WGS result. Ten to 20 times higher SNP rate than average FLA SNP rate was found in class II DR region, class II/III border region, proximal class I region and pericentromeric and subtelomeric regions. Clustered high coding SNP rates were observed in the proximal class I region.

**Table 4:** Characterization of 19 Class I Genes in *FLA*.

Methods Applied							
Gene	Dotplot^a^ with full length cDNA	Coding Prediction		Sequence^d^ homology with cDNA	31 conserved^e^ Amino acid residues in α1/α2 domains	Assignment^f^	*BAT1p* g association
		I^b^	II^c^				
*FLA I-A*	+	−	−			nonclassical	−
*FLA I-B*	−					gene fragment	−
*FLA I-C*	+	−	−			nonclassical	−
*FLA I-D*	−					gene fragment	−
*FLA I-E*	+	−	++		++ (All)	classical	+
*FLA I-F*	+	−	++		− (−5)	nonclassical	−
*FLA I-G*	−					gene fragment	−
*FLA I-H*	+	+	+	+	+ (−1)	classical	+
*FLA I-I*	−					gene fragment	−
*FLA I-J*	+	+	+		− (−3)	nonclassical	+
*FLA I-K*	+	+	++	+	++ (All)	classical	+
*FLA I-L*	+	−	+		− (−5)	nonclassical	+
*FLA I-M*	+	−	+		− (−6)	nonclassical	+
*FLA I-N*	−					gene fragment	−
*FLA I-O*	+	+	−		− (−5)	nonclassical	+
*FLA I-P*	−					gene fragment	+
*FLA I-Q*	+	−	−			nonclassical	−
*FLA I-R*	−					gene fragment	−
*FLA I-S*	+	−	−			nonclassical	−

a. PIPmaker dotplot ( ) was used. + and − represent full-length and partial length, respectively compared with full length FLAIA24 cDNA.

b. GENSCAN was used to predict coding region for only full-length class I genes. + and − symbols represent right and wrong prediction of exon and intron boundaries in each gene.

c. Spidey was used to examine sequence alignment of genomic cDNA class I sequences and splicing donor/acceptor sites. ++, +, and − symbols represent typical class I exon/intron structures reported in human class I genes with all correct splicing donor/acceptor sites, with one or two missing splicing donor/acceptor sites, and atypical exon/intron structures, respectively.

d. Class I cDNA sequences from MHC homozygous feline fibroblast cells were compared with all class I genomic sequences by Megablast Search ( ). + symbol represents >99% sequence identity.

e. Thirty-one highly conserved amino acid residues found in α1 and α2 domains of human and cat class I antigens were examined. ++, +, − numbers represent all 31 conserved residues, one substitution and more than one substitutions, respectively.

f. Assignment of classical/nonclassical/gene fragment class I genes based on this study.

g. Symbols + and − represent presence and absence of *BAT1*gene fragment in vicinity of class I gene.

### Two DR haplotypes in a single BAC library

**Figure 6:**
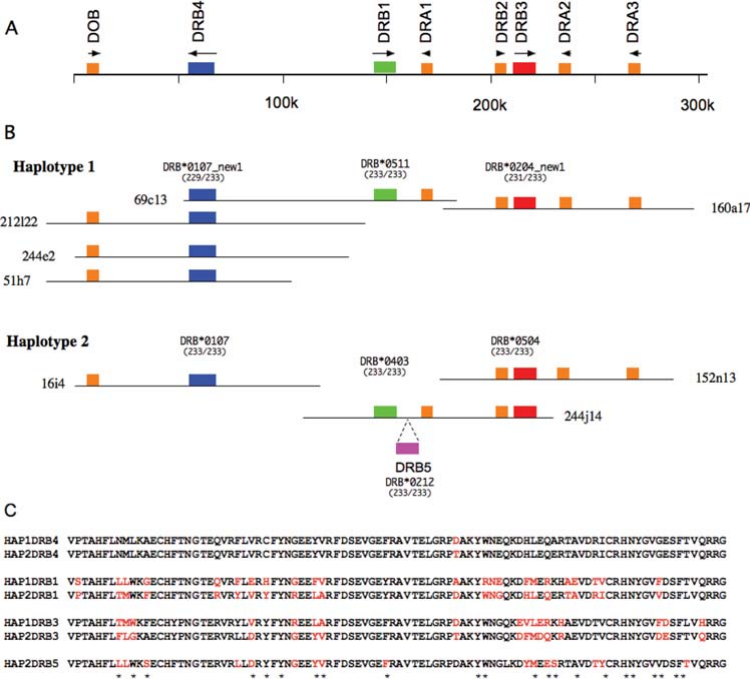
Haplotype analysis of the domestic cat MHC class II DR region. (A) Gene organization of the domestic cat MHC class II DR region based on the nucleotide sequence of a composite haplotype as previously reported in Yuhki *et al.* [14]. The location of eight DR genes is shown with the transcriptional orientation indicated by arrows. (B) Analysis of the haplotype structure of Fca273 (used to make the BAC library) based on mapping of gene content of individual BAC clones by hybridization and sequence-based typing of exon 2 of BAC clones. DRB alleles were identified based on comparison to 71 domestic cat DRB exon 2 sequences spanning 233 bp (after removal of primer sequences) present in the NCBI nucleotide database. (C) Deduced amino acid sequences from two haplotypes were aligned in each loci and different residues in each loci were depicted in red. Antigen recognition sites were shown as asterisks below the sequence alignment.

**Figure 7:**
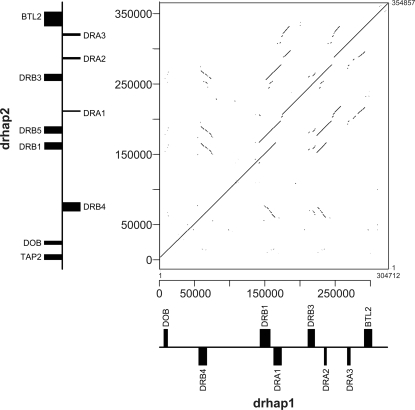
Dotplot analysis of two DR haplotype sequences from a single BAC library.

We previously constructed a composite nucleotide sequence of the domestic cat MHC class II region spanning 758 kb from HSET to BTLII that included the DR region [18]. As shown in Figure 6A the DR region spans approximately 250 Kb and consists of 3 DRA and 4 DRB genes, both gene families are encoded by 5 exons with the exception of DRB2 which lacks the full complement of exons and thus represents a pseudogene. We determined the sequence of the second haplotype of a domestic cat DR region using BAC clones (152n13–244j14–16i4) from a single individual. The DR haplotype 2 contained three complete DRA genes (DRA1, DRA2 and DRA3), five DRB genes (four complete and one partial) similar to DRB4, DRB1, DRB3 and DRB2 plus new DRB gene, namely DRB5 as well as a BTLII gene all with the same order and orientation as observed in the DR haplotype 1 (except DRB5 adjacent to DRB1 with same orientation) (Figure 6B). To determine if DRB1 and DRB4 also displayed allelic variation, we aligned the genomic sequences of DRB1, 3 and 4 in the region of exon 2 and flanking introns 1 and 2. To assign these exon 2 sequences to specific DRB alleles we compared them to 71 different domestic cat DRB exon 2 alleles of 238 bp in length present in the NCBI nucleotide sequence database (nr/nt). The results summarized in Figure 6B show that DR haplotype 2 contains DRB3 exon 2 identical to DRB*0504 whereas DR haplotype 1 contained a DRB3 exon 2 that differed by 2 bp from DRB*0204, and thus represents a new domestic cat DRB allele (DRB*0204_new1) but differ from nucleotides of 70 nts between DR hap1 and DR hap2. Similarly, DRB1 exon2 sequences in haplotypes 1 and 2 contain the alleles which were identical to DRB*0511 and DRB*0403, respectively, but differ in 31 nucleotides of 233 nts. In addition, haplotype 1 was also positive for DRB4 which showed 229/233 nucleotide sequence identities with DRB*0107_new1. In contrast, the haplotype 2 DRB4 sequence was identical (233/233) to DRB*0107. The DR haplotype 2 contains additional DRB genes designated DRB5 that was not observed in haplotype 1 that displays identical exon 2 sequences with DRB*0212. In summary, this data show that a single domestic cat (Fca273) contains three or four DRB genes in the order DRB4-DRB1-(DRB5)-DRB3, that the three loci are heterozygous, and resolve into 2 distinct haplotypes consisting of DRB*0107_new1-DRB*0511-DRB*0204_new1 (haplotype 1) and DRB*0107-DRB*0403-DRB*0212-DRB*0504 (haplotype 2). Dotplot analysis confirmed this conclusion, indicating duplicated DRB genes adjacent to DRB1 gene (Figure 7). Deduced amino acid sequences of above alleles were compared in each DRB loci (Figure 6C). Of these loci, DRB1 alleles were the most polymorphic containing 23 different residues. DRB3 was the second most polymorphic loci, maintaining 17 different residues. In contrast, DRB4 had only one amino acid substitution.

The additional DRB loci found in haplotype 2, named as DRB5 had 16 different residues compared with DRB1 loci in haplotype 2. On antigen recognition site (ARS) defined by X-ray crystallography [36], 22 sites forms ARS. Of these sites, 15 sites were found highly polymorphic in FLA.

### Transcription factor (TF) binding sites in predicted classical genes

**Figure 8:**
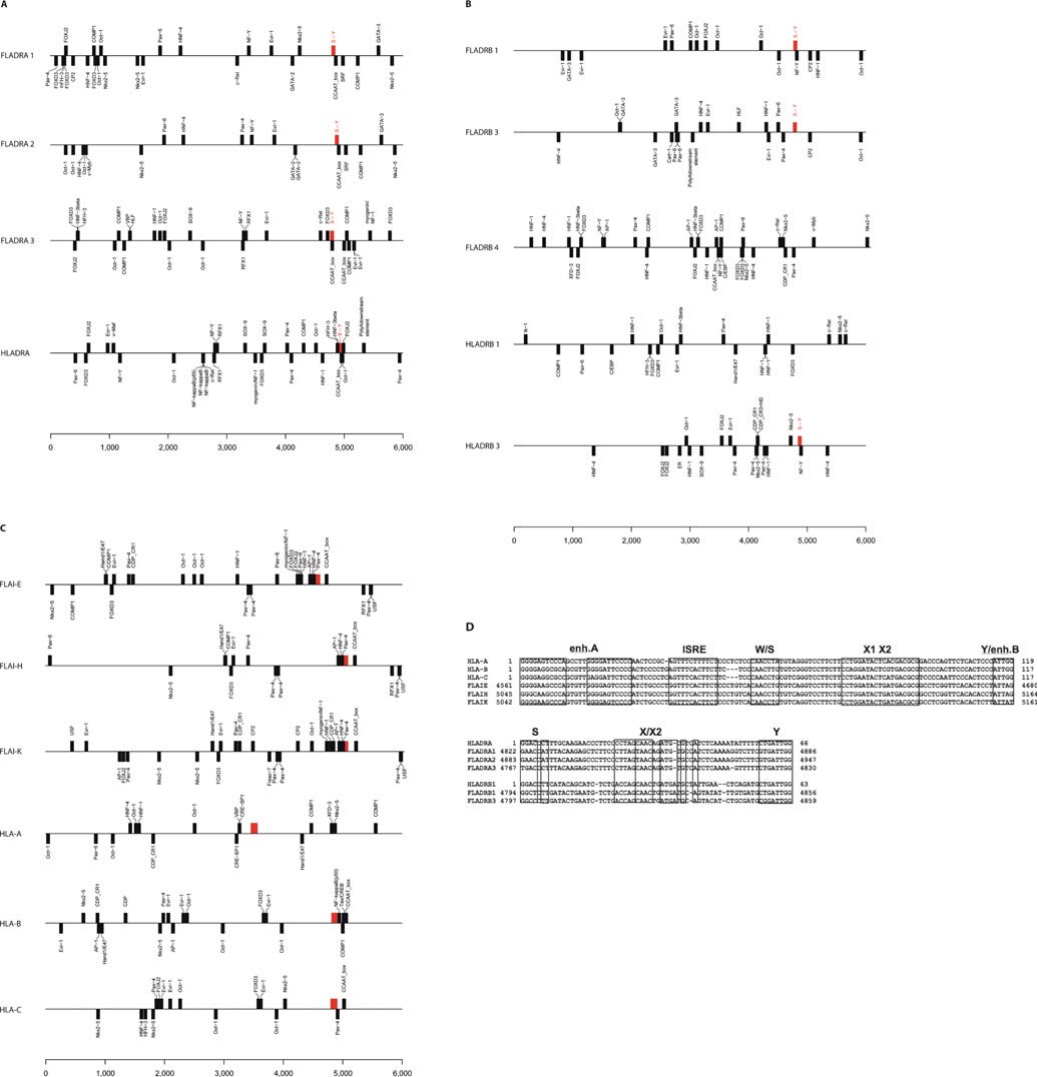
Transcriptional factor binding site prediction. A total of 6 kb sequence spanning 1 kb downstream and 5 kb upstream from translation start site (ATG) were analyzed for (A) DRA genes, (B) DRB genes, and (C) Classical class I genes. The S-X/X2-Y module and enh.A-ISRE-W/S-X1-X2-Y.enh.B were depicted as a red box. HLA-DRB1, FLA- DRB4 modules were located at 52 Kb, 7 Kb upstream from ATG site, respectively. Forward and reverse orientation of TF binding sites were depicted above and below lines respectively: (D) enh.A-ISRE-W/S-X1-X2-Y/enh.B module sequences found in FLA-E, -H, -K and HLA-A, -B, -C genes and S-X1-X2-T module sequences found in FLA-DRA1, -DRA2, -DRA3, -DRB1, -DRB3 and HLA-DRA, -DRB1, were aligned and each promoter/enhancer cis-motifs were boxed. Coordinates of FLA were based on 6 Kb sequence described above.

We have analyzed transcription factor binding sites a total of 6 kbp (5 kb upstream and 1 kb downstream of ATG putative translation start site) of predicted feline classical class II genes (DRA1, 2, 3, DRB1, 3, 4: Figure 8A, Figure 8B) and classical class I gene candidate genes (I-E, I-H, I-K: Figure 8C), plus human classical class II and I genes (DRA, DRB1, DRB3, HLA-A, -B, -C). Figure 8A depicts the result of DRA genes. All three feline DRA genes have CCAAT-box. The DRA1 and DRA2 genes have striking similarity with TF binding sites up to about 4 kb upstream of ATG site and at least NF-Y binding site, indicating recent gene duplication. In contrast, the DRA3 gene has distinct TF binding sites from the other two genes and is relatively similar to those of the human DRA gene, (e.g., NFY-RFX1-RFX1 sites, Oct-1 sites, sox-9 sites). It may be suggested that the expression pattern is different in these two groups of DRA genes.

Figure 8B depicts the difference between TF binding sites in feline DRB1, -3, -4 genes, and human DRB1 and -3. All of these genes lack the CCAAT-box site. The NF-Y site was found in FLA-DRB1, DRB4, and HLA-DRB3. However, no apparent similar TF binding site patterns were found. In class I genes, all predicted feline classical class I genes have a CCAAT-box site plus a unit of AP1-HNF4-Pax-4 sites adjacent to the CCAAT-box (Figure 8C). FLA I-H, I-K had relatively similar TF binding sites (e.g., Pax-4-Pax4, Evi-1, FOXD3-COMP1-Hand1/E47, Nkx2-5). Human HLA-B, -C had relatively similar TB binding sites (e.g., CCAAT-box, Oct1, Evi-1-FOXD3, Evi-1) however, the HLA-A gene had no CCAAT-box and was quite different in TF site pattern from HLA-B, -C genes. No apparent similar TF binding patterns were found in the FLA and HLA classical class I genes.

MHC class I and class II gene promoter structures were well documented and intensely analyzed by many molecular biological methods [37], [38]. MHC class II genes are regulated by a complex system containing two gene-specific transcription factors, regulatory factor X complex (RFX) and CIITA, and maintain an approximately 67 bp sequence, a strictly conserved regulatory module (S-X1-X2-Y) immediately upstream of the promoters [37]. In contrast, MHC class I genes are regulated by NFκB2, NFκB1, interferon-γ, RFX, and CIITA, and form an approximately 120 bp conserved regulatory module sequence, enh.A-ISRE-W/S-X1-X2/site α-Y/enh.B [38].

Similar conserved regulatory modules were identified in most of FLA class I and II genes analyzed here and summarized in Figure 8D.

## Discussion

We report here annotation and SNP analysis of cat MHC (FLA). This study revealed one hundred forty-seven human gene homologues with mostly conserved gene order in five subregions, extended class II, class III, proximal class I, central class I, and distal class I regions. Extensive rearrangement events were obvious in classical class II and class I regions by dotplot analyses of three mammalian MHC, human HLA, canine DLA, and feline FLA (Figure 3). Especially, deletion of HLA-A and -E regions in both DLA and FLA, and expansion of the regions in FLA corresponding to HLA-B, -C were clearly observed (Figure 3A, B). A dotplot between DLA and FLA (Figure 3C) suggests that these two MHC systems are more syntenic than those to HLA. However, the manner of class I rearrangement was unique in each DLA and FLA. Each DLA and FLA also had unique sequences near the heterochromatin regions (near telomere and centromere) in canine chromosome cfa35ter/cfa12cen and feline chromosome B2pter/qcen regions. Among mammalian and MHC class I regions reported so far, only mammals which belong to the group Euarchontoglires (Primates and Rodentia) have class I E and A subgroups, plus the evidence of the recombinant origin of the class I E gene between class I A and B/C [39] suggests that the formation of these two class I subgroups (A, E) occurred after the split of two major mammalian groups, Euarchontoglires and Laurasiatheria [Carnivora (dog and cat), Perissodactyla (horse), Certartiodactyla (pig and cattle)].

### Class II genes in FLA

Unlike all other mammalian MHCs which have a single DRA gene, FLA maintains three possible functional DRA genes due to two possible duplication events and one inversion [18]. The deduced amino acid sequences coding a mature DRA peptide are identical in these three DRA genes. However, significant levels of difference in amino acid sequences in the signal peptide region, which may suggest distinct roles in this region. In addition, distinct TF binding sites in DRA1/2 and DRA3 may suggest distinct expression patterns. All three DRB genes, common in two haplotypes examined had significant levels of polymorphism in exon 2 sequence which encodes peptides forming antigen binding and T cell receptor recognition sites. The well documented S-X1-X2-Y promoter module sequences were found in all DRA and DRB genes immediate upstream of CCAAT-box site , except that DRB4, which maintains this module sequence 7 kb upstream from ATG site and 5.5 kb upstream of CCAAT-box. FLA is also unique among mammalian MHC due to the fact that the entire DQ region is deleted. Since canine MHC (DLA) maintains a pair of A and B genes in its DQ region, this deletion event may occur after the split of canids and felids (55MYA).

### Class I genes in FLA

Class I gene amplicon of a combination of class I and BAT1 gene fragments are found here in FLA-specific manner, though the human HLA-A region has two BAT1 gene fragments, suggesting that relatively new origins of multiple class I genes than classical class II families (DP, DQ, DR), which were estimated more than 80 MYA [40]. Gene structure of 19 FLA class I genes was characterized and summarized in Table 5. Eleven class I genes maintained full-length exons by dotplot, when compared with FLA class I cDNA sequence and their coding sequences were predicted by GENSCAN. Of those, six class I genes had intact splicing donor/acceptor sites. Three genes (FLA I-E, I-H, I-K) had 31–32 highly conserved amino acid residues in α1 and α2 domains which were reported in deduced amino acid sequences of FLA class I transcripts from fibroblast cell lines [41]. Analysis of FLA class I transcripts of a fibroblast cell line from MHC homozygous Abyssinian cat used for cat genome project indicated that these transcripts are derived from FLA I-H and I-K. In addition, all three of these class I genes maintain the conserved enh.A-ISRE-W/S-X1/X2-Y/enh.B promoter motif immediately upstream of CCAAT-box. Together, we tentatively assigned FLA I-E, I-H, I-K as classical class I genes and nine other genes as nonclassical class I genes.

**Table 5:** Single Nucleotide Polymorphism (SNP)s.

	*FLA*	*HLA*
Size (Mbp) compared	2.84 Mbp	4.75 Mbp
No. of SNPs	11,654	16,013
SNP rate (per bp)	0.00411	0.00337
No. of CDS SNPs	732	341
Class I & II genes S/N	48/145	48/68

This promoter analysis also revealed potentially distinct gene regulation of other FLA class I genes. For example, FLA I-S and I-O genes had an intact conserved promoter motif. However, I-S class I gene did not have intact coding region nor expression in fibroblast (Table 5). Also I-O class I gene did not maintain 5 highly conserved deduced amino acid residues in its peptide binding groove. Other class I genes, FLA I-A, I-Q lacked NFκB1, 2 and IFN-γ binding sites, and FLA I-J, I-L lacked Y/enh.B site.

### Single Nucleotide Polymorphism (SNP)s

Overall, the SNP rate found in FLA (BAC sequence versus MHC homozygous 1.9× WGS contigs) was at least twice as much higher than the SNP rate in average heterozygous region in the WGS cat genome, (0.00411 versus 0.0017) and slightly higher but nearly equivalent to the SNP rate found in two human HLA haplotypes (6COX and 6QBL) (Table 4). A total number of coding SNP (CDS SNP) is higher than human HLA (732 versus 341). A total of 193 CDS SNPs were found in class II and class I genes. Of these, both class II DRB4 and DRB1 genes had a higher number of nonsynonymous CDS SNPs than synonymous ones, and two class I genes (FLA-I, -F, -H) had similar tendencies (Table 6). These data suggest that those genes are under positive selection.

**Table 6:** Nonsynonymous and Synonymous Coding SNPs in *FLA* class I and II genes.

Class	*FLA* class I/II genes	No.of Synonymous and Nonsynonymous SNPs (S/N)
II	*DRB4*	8/25
II	*DRB1*	1/2
II	*DRB3*	5/13
II	*DRA1*	2/0
II	*DRA2*	0/0
II	*DRA3*	0/0
I	*I-A*	1/0
I	*I-C*	2/1
I	*I-E*	0/0
I	*I-F*	4/40
I	*I-H*	15/50
I	*I-J*	6/7
I	*I-K*	0/1
I	*I-L*	1/1
I	*I-M*	0/1
I	*I-O*	3/4

### New Endogenous Retrovirus Sequences

Phylogenetic analysis of three FLA endogenous retrovirus sequences using the neighbor-joining method (Figure 4) suggested that in addition to previously described baboon-derived RD114 retrovirus (or ECE1) [42]–[44] the other two sequences showed equidistance to FeLV derived [45], [46] and RD114 endogenous sequences, but more similar to the sequence recently submitted to GenBank as comparative genome initiative research derived from brown bat (Myotis Lucifugus) BAC sequence. Because all sequences described above maintained retrovirus POL region, newly identified feline retrovirus sequences was assigned as FERVmlu1 (previously FERV1) and FERVmlu2.

### MHC Class I Related Genes

Of four MHC class I-related genes (MIC) which encodes c-lectin type NK receptor ligands in HLA, none of them had full length exon sequences when compared with human MICA transcripts (data not shown). Interestingly, neither cat nor dog genomes maintain multigene families of KIR and Ly49 found in primates and rodents genomes, respectively. These evidences may suggest distinct control systems for NK cells in cats and dogs.

### Comparison of genomic structures in cat, dog, human, mouse, and opossum MHC genes

**Figure 9:**
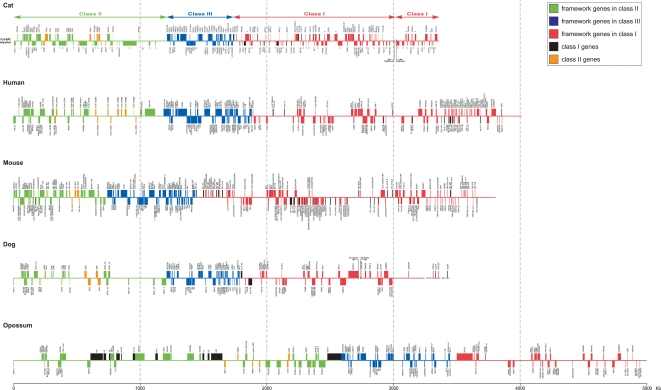
Comparisons of MHC genomic structures in cat, human, mouse, dog, and opossum. Framework genes in class II, III, I regions were shown as green, blue, red boxes, respectively. Forward and reverse orientations of each gene were shown above and below line, respectively. Classical class II antigen coding genes/gene fragments were shown in orange and classical and non-classical class I genes were shown in black.

A proportionally scaled MHC genomic structure was presented for four mammalian genomes (cat, human, mouse, and dog) and one marsupial genome (opossum) (Figure 9). The MHC region spanning from KIFC (except mouse H2 which has a translocation in this region, so that H2 here compared from Rps 28) to UBD plus 3 olfactory receptor genes was compared in these MHCs. The result depicts striking similarity in gene contents and order of framework genes from marsupial through mammalian evolution. Three MHC (opossum, human, and mouse) have one contiguous gene content, suggesting depiction of an ancestral form of MHC, while two MHC (cat and dog) have a same split form of MHC at TRIM31 and TRIM26 in the class I region as compared with human HLA. In dog MHC, these two pieces were located on two chromosomes (cfa12qcen, cfa35qter), while in cat MHC, these were located on a single chromosome by an inversion (FcaB2 qcen, FcaB2pter) as previously described [19]. Further, two class I genes in dog MHC were located with two additional chromosomes (cfa7, cfa18). The size variation of MHC from about 3.3 Mbp (cat and dog excluding percentromeric, subtelomeric regions) to about 5 Mbp in opossum was also seen in this analysis. The difference in size observed here is mainly due to the magnitude of class I gene amplification and size of class II/III border regions. Cat MHC consists of 650 Kbp class I gene region, spanning from BAT1 to POU5F1 maintaining 17 class I genes/gene fragments, while human and dog MHC have only 2–3 class I genes in this region. Mouse H2 has 7 class I genes and there are no class I genes in opossum MHC in this region. Accordingly, class I gene amplification seemed to have occurred in a species-specific fashion. Additional evidences that e.g. opossum MHC, class I genes were amplified in the class II region, human HLA have at least 11 class I genes in the HLA-A region between the ZNRD1 and MOG genes and in mouse H2, at least 15 class I genes were found between Abcf1 and Trim26 genes, all support adaptive evolution of this importance immune system. Interestingly, the sizes of class II/III border regions vary in each MHC. Cat and dog MHC have approximately 400 Kbp in these regions. In cat MHC, this region was occupied with LINE repeats however, in dog and opossum there are multiple BTNL genes. These evidences reaffirmed the dynamic nature of evolution and maintenance of genome organizations in MHC.

## Supporting Information

Figure S1 (3.98 MB BZ2)
