# Supplementary figures and images for: Sequences, Annotation and Single Nucleotide Polymorphism of the Major Histocompatibility Complex in the Domestic Cat
Source: PLoS One. 2008 Jul 16;3(7):e2674. doi: 10.1371/journal.pone.0002674 (PMC2453318; doi:10.1371/journal.pone.0002674)

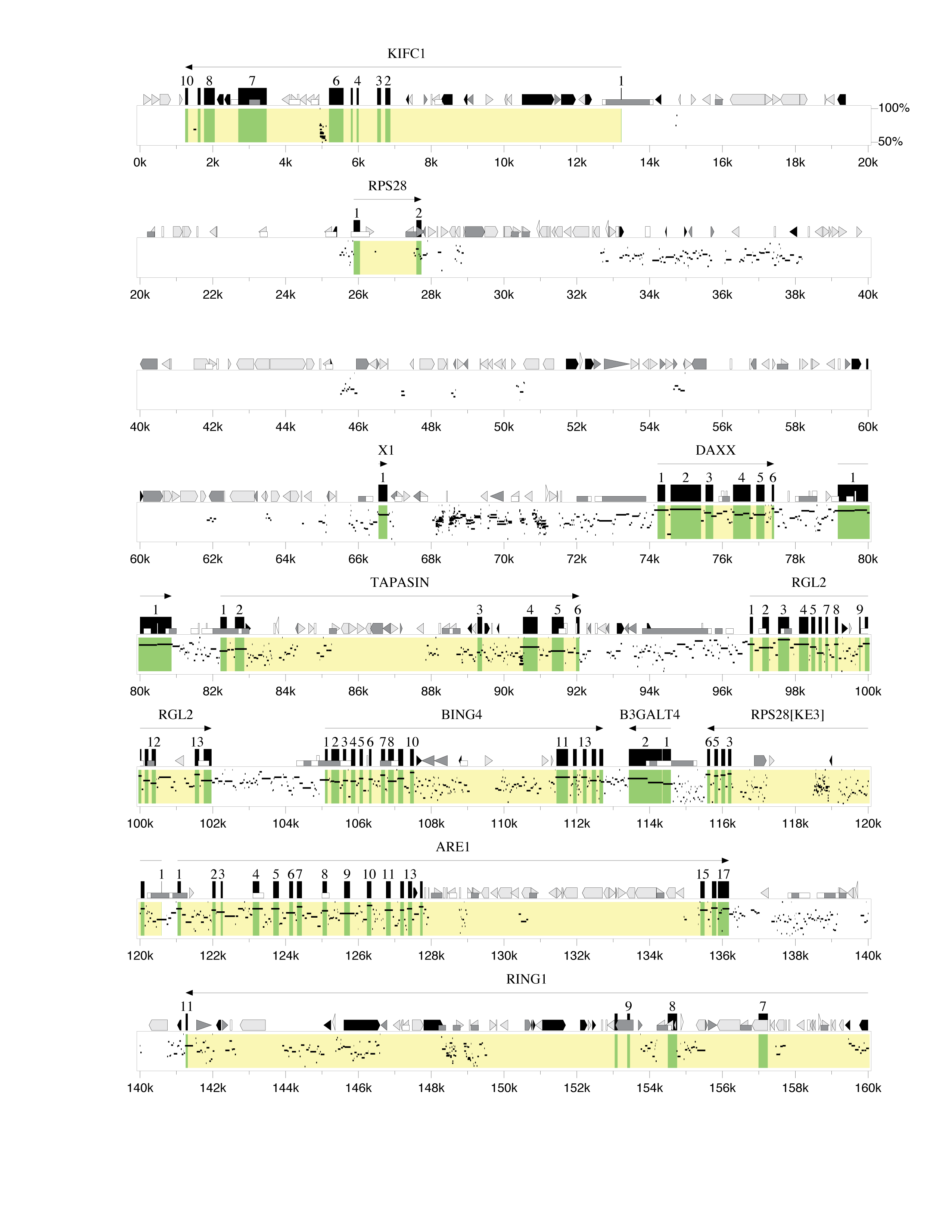

Supplement: Figure S1 — (3.98 MB BZ2) [file pone.0002674.s001.bz2 › Figure2A_1.png]

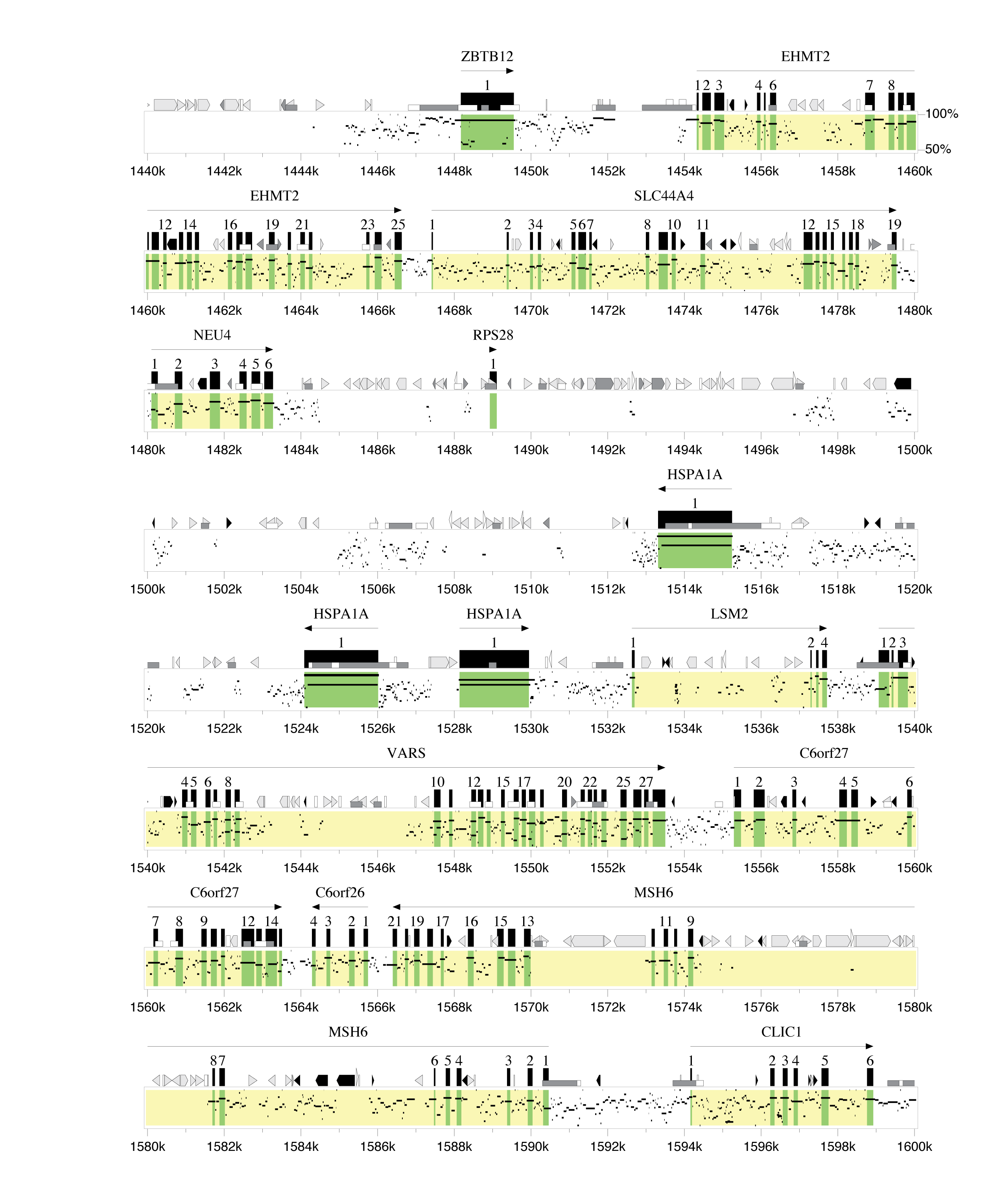

Supplement: Figure S1 — (3.98 MB BZ2) [file pone.0002674.s001.bz2 › Figure2A_10.png]

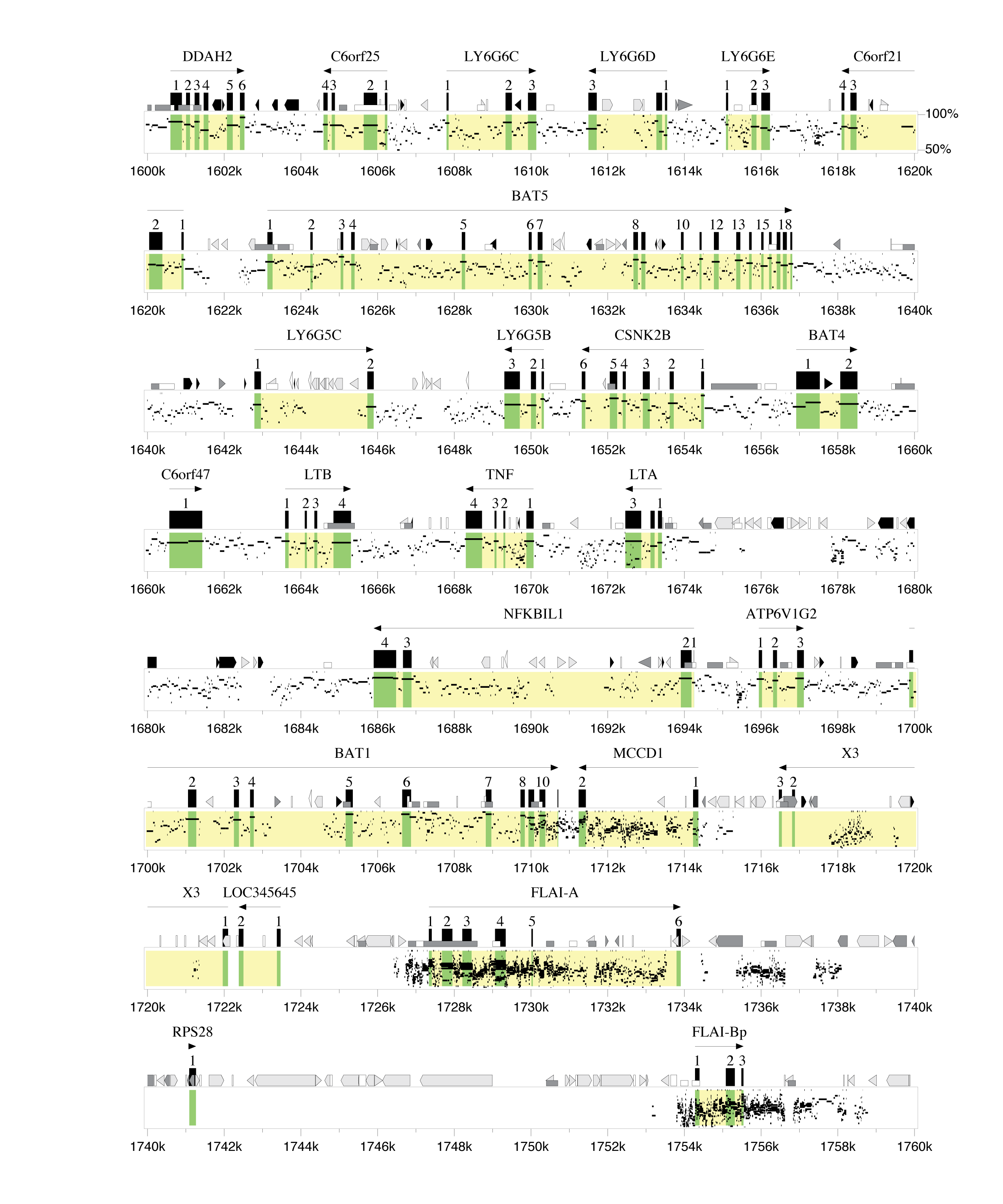

Supplement: Figure S1 — (3.98 MB BZ2) [file pone.0002674.s001.bz2 › Figure2A_11.png]

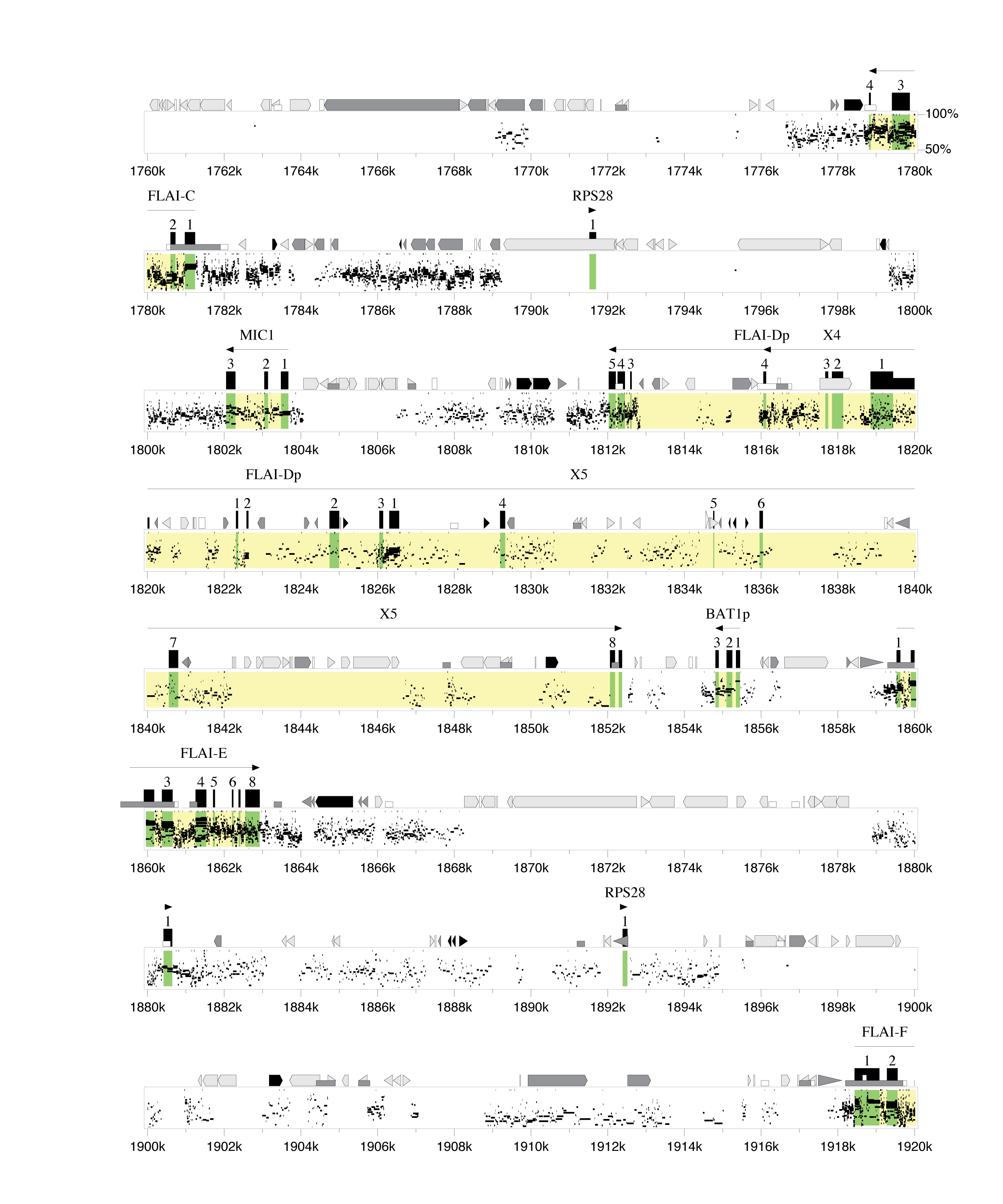

Supplement: Figure S1 — (3.98 MB BZ2) [file pone.0002674.s001.bz2 › Figure2A_12.png]

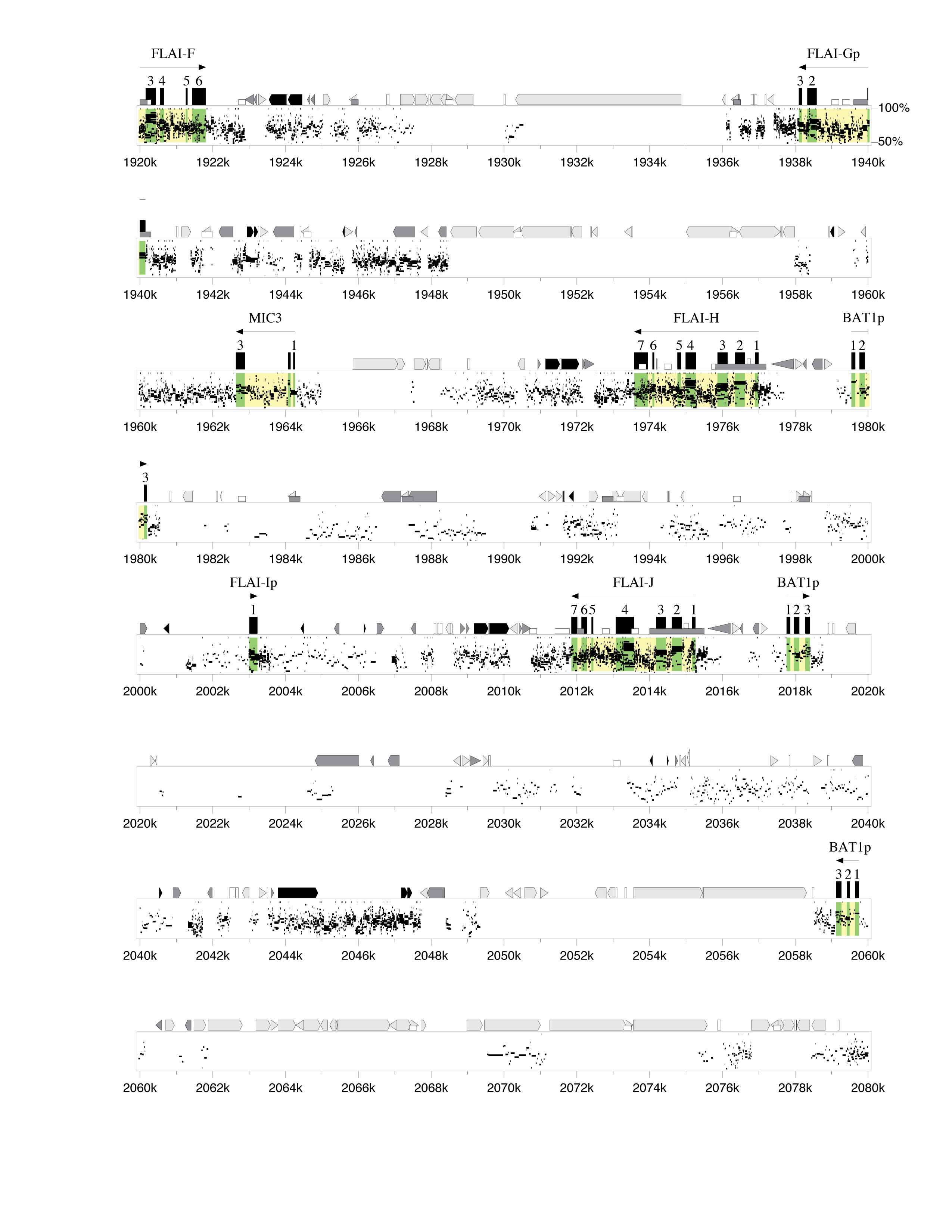

Supplement: Figure S1 — (3.98 MB BZ2) [file pone.0002674.s001.bz2 › Figure2A_13.png]

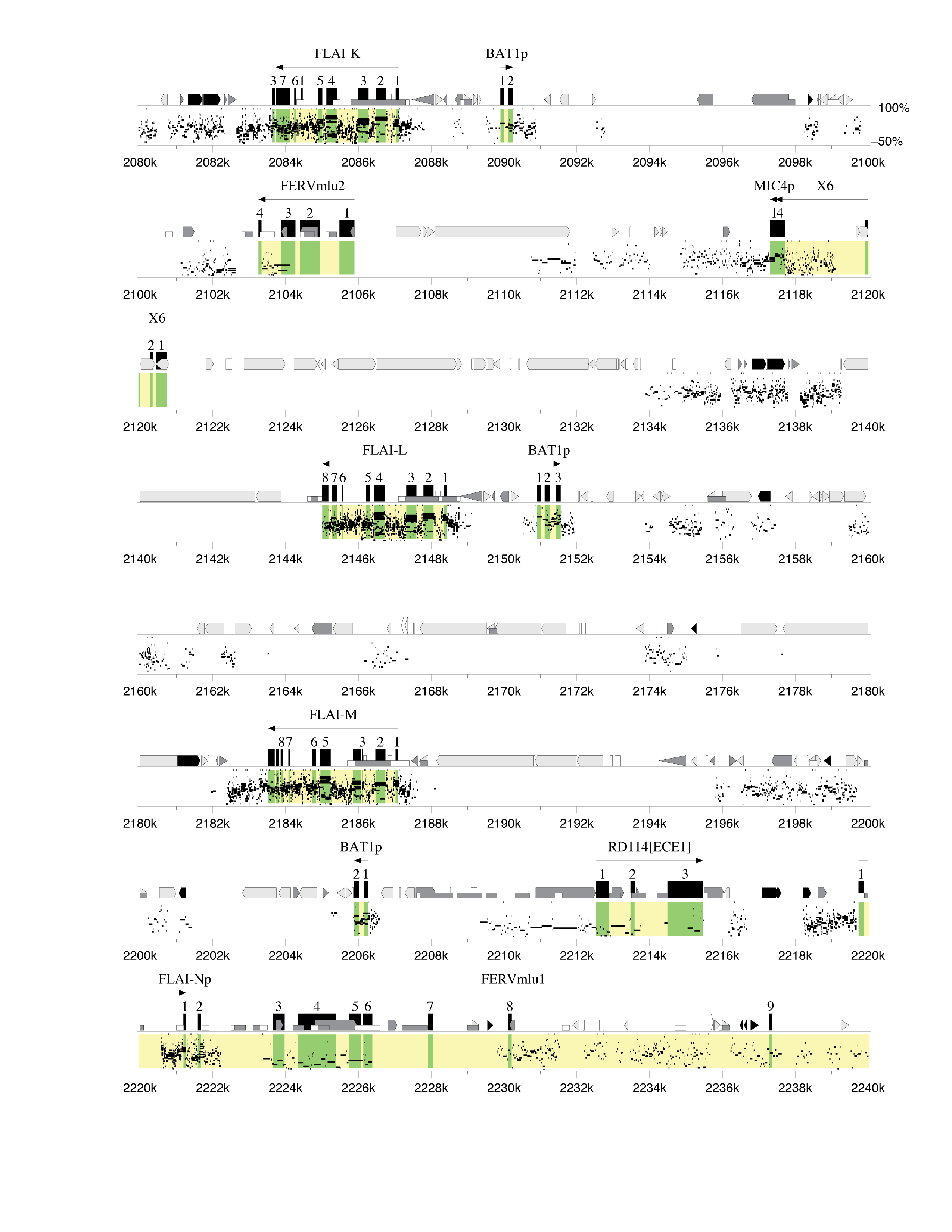

Supplement: Figure S1 — (3.98 MB BZ2) [file pone.0002674.s001.bz2 › Figure2A_14.png]

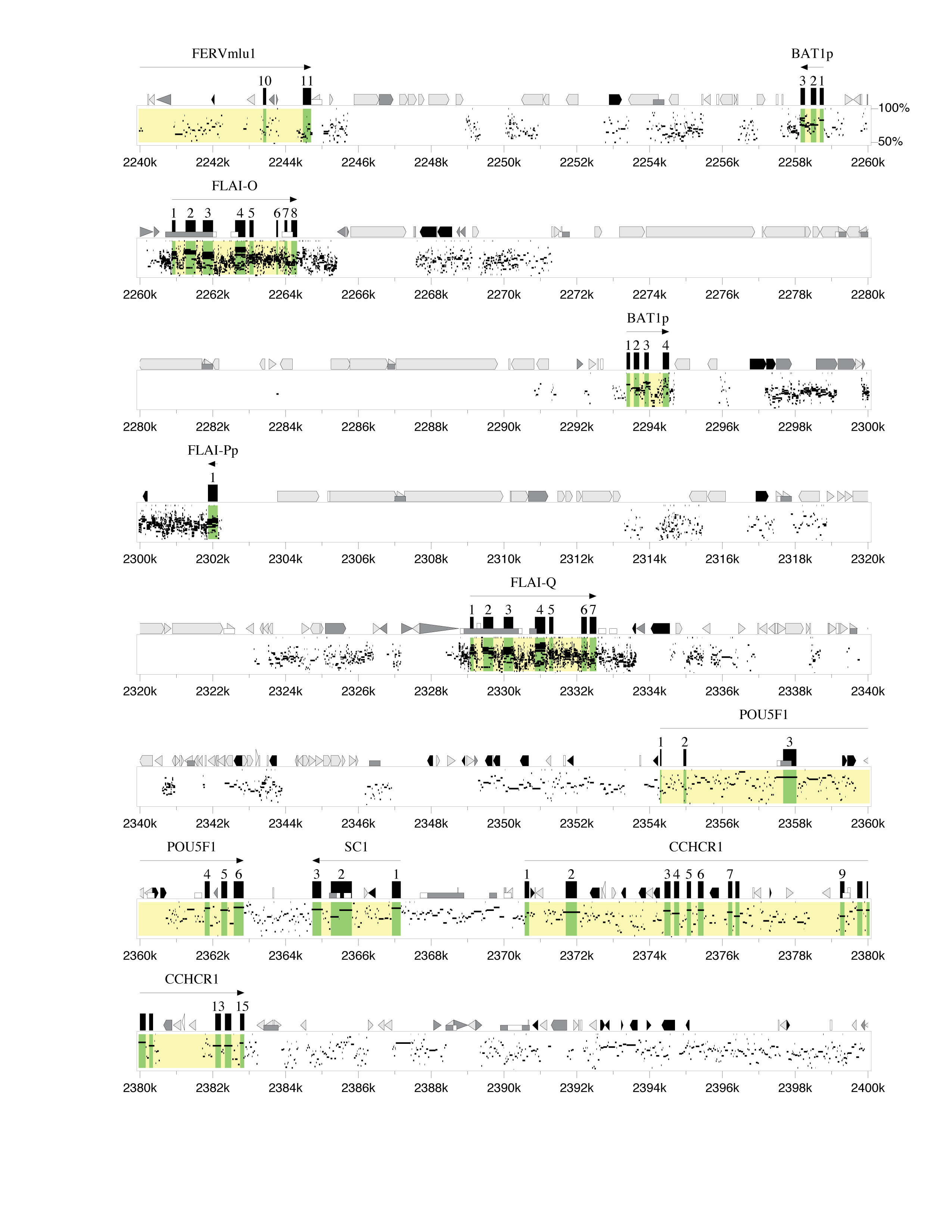

Supplement: Figure S1 — (3.98 MB BZ2) [file pone.0002674.s001.bz2 › Figure2A_15.png]

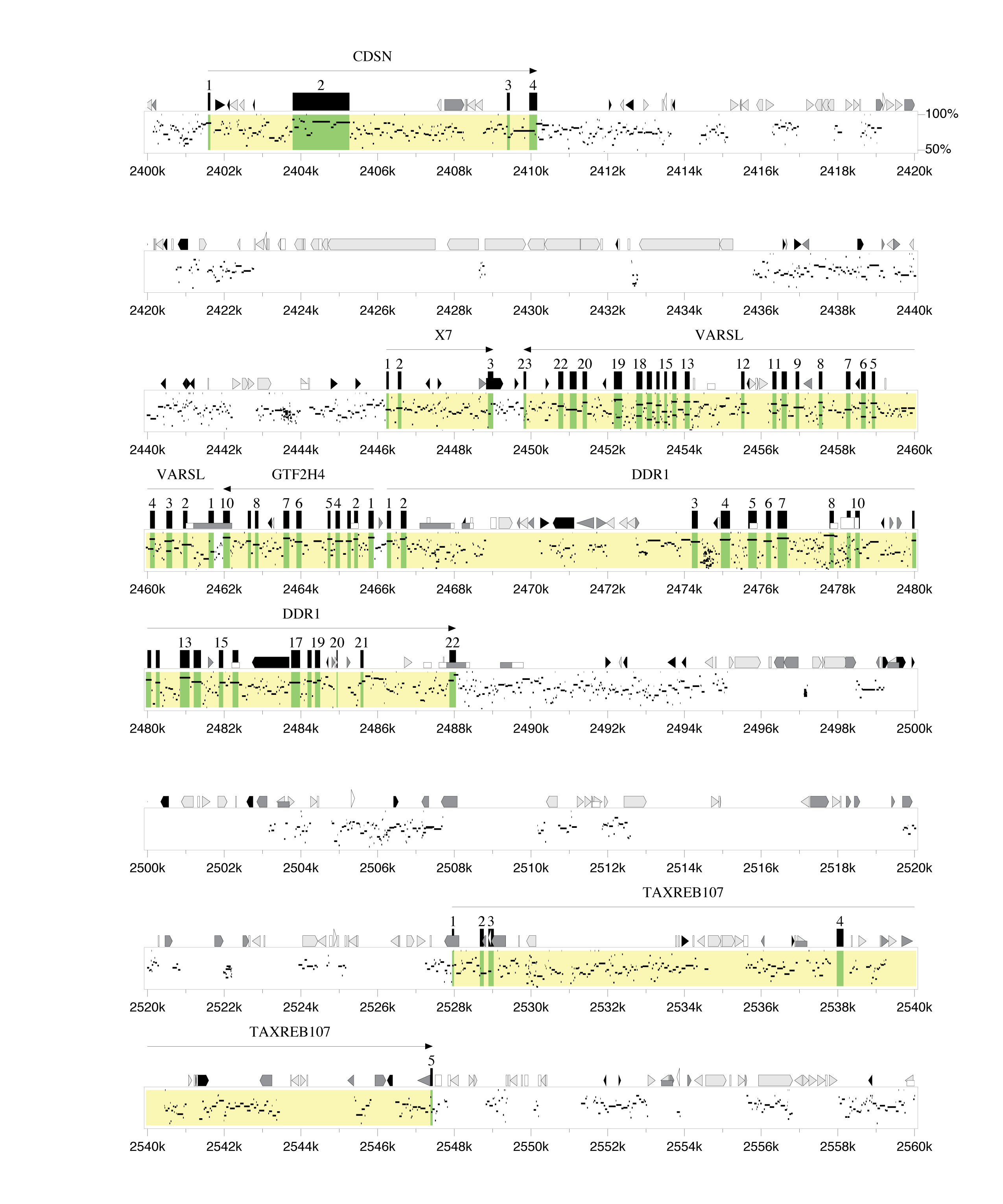

Supplement: Figure S1 — (3.98 MB BZ2) [file pone.0002674.s001.bz2 › Figure2A_16.png]

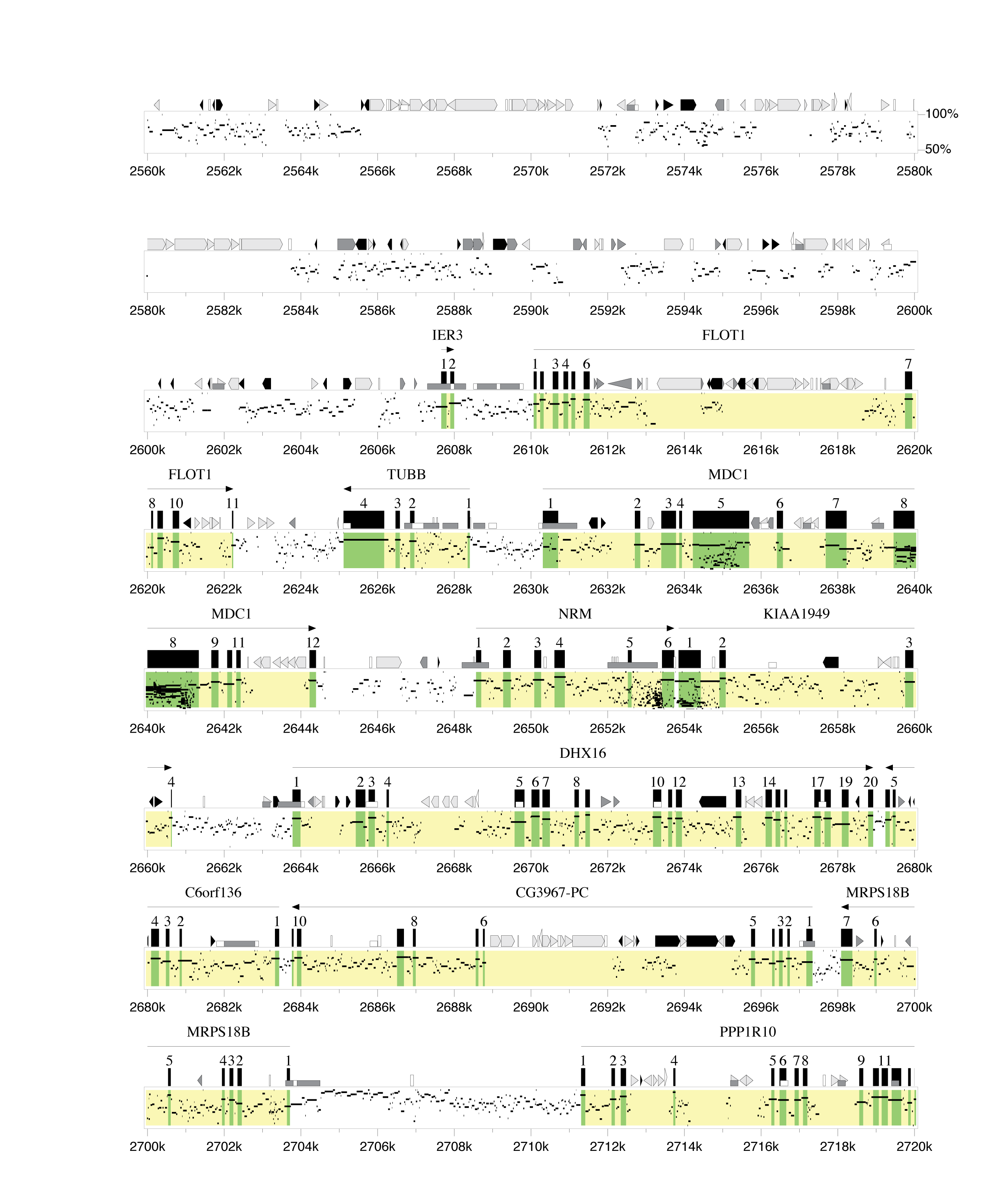

Supplement: Figure S1 — (3.98 MB BZ2) [file pone.0002674.s001.bz2 › Figure2A_17.png]

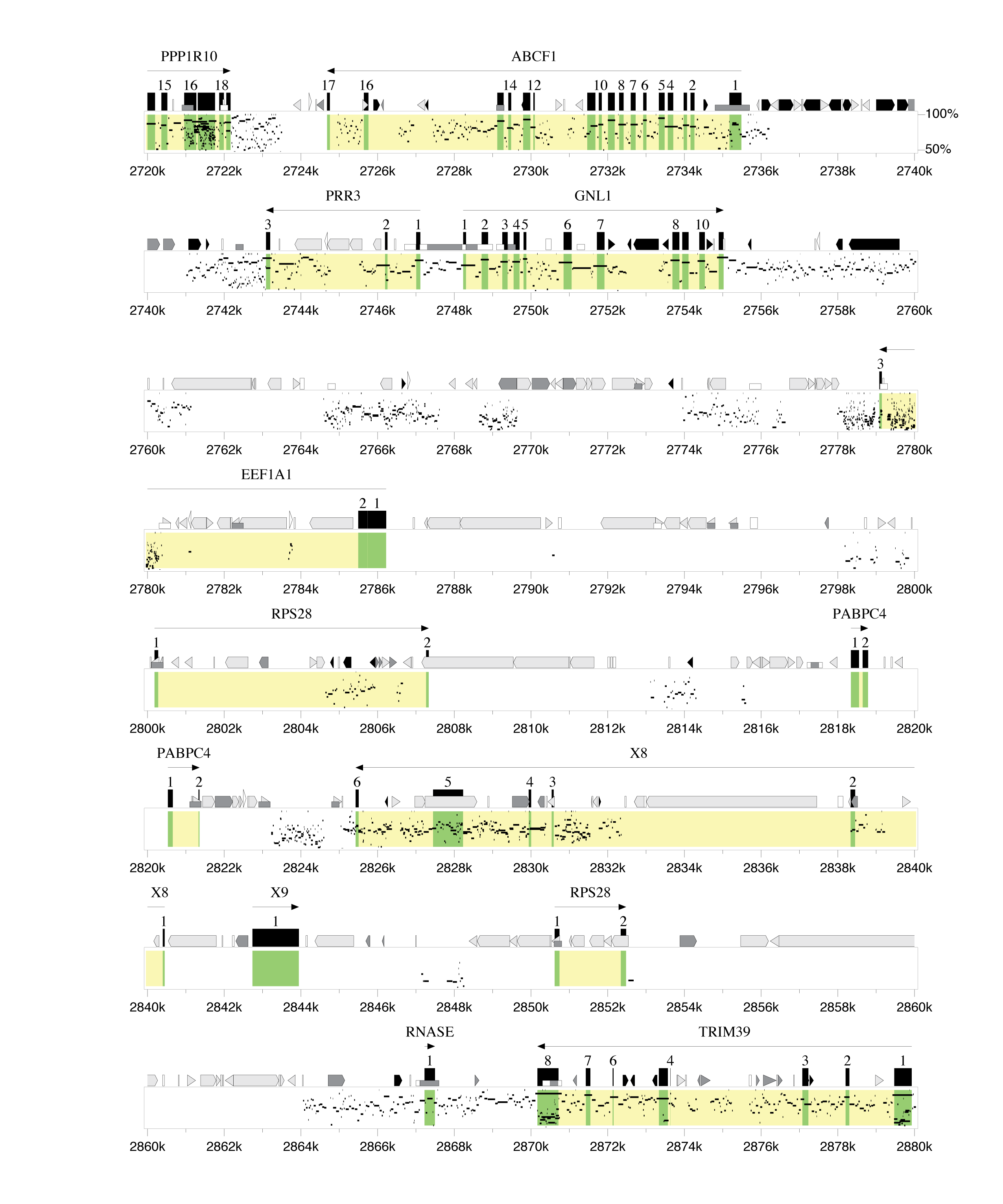

Supplement: Figure S1 — (3.98 MB BZ2) [file pone.0002674.s001.bz2 › Figure2A_18.png]

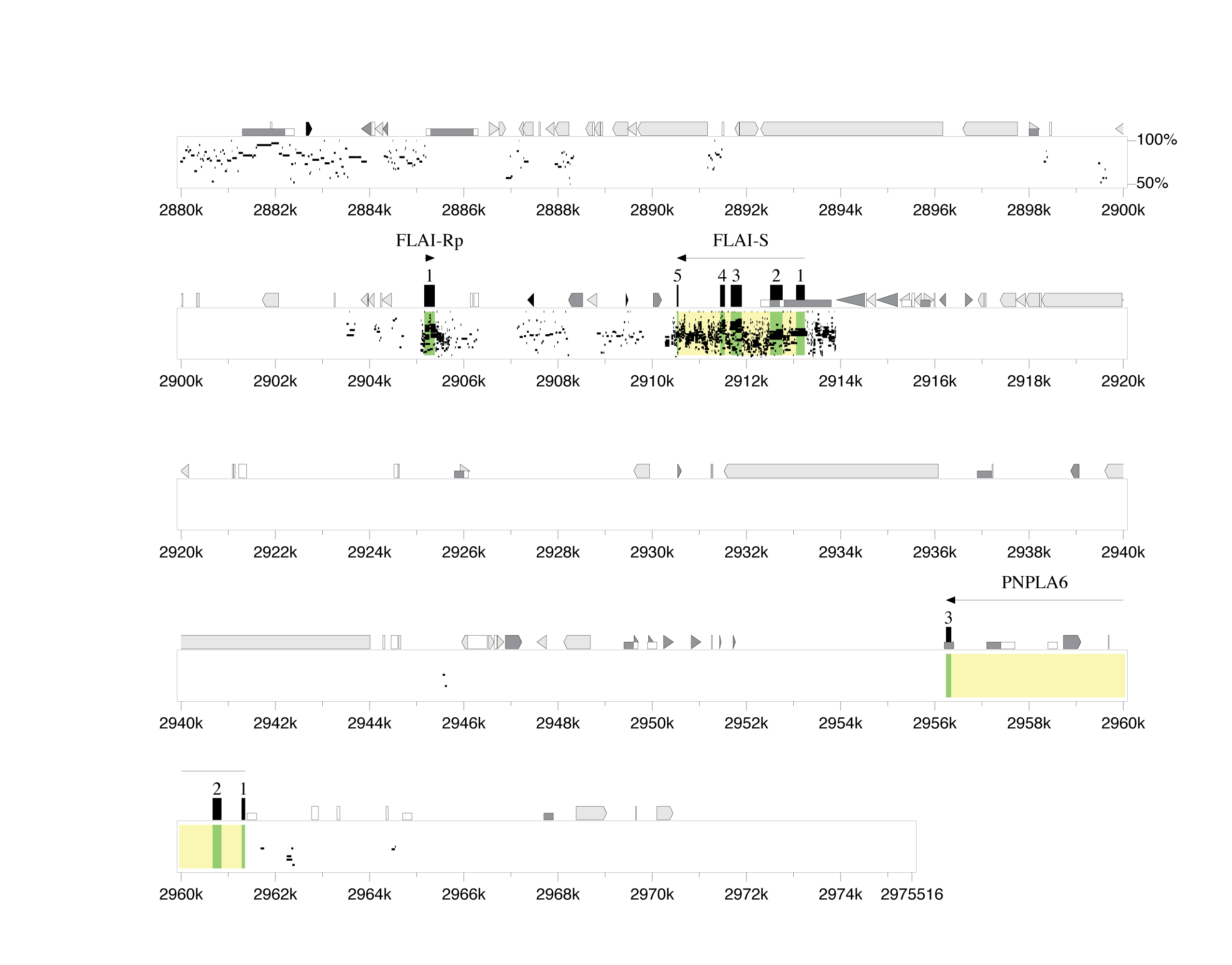

Supplement: Figure S1 — (3.98 MB BZ2) [file pone.0002674.s001.bz2 › Figure2A_19.png]

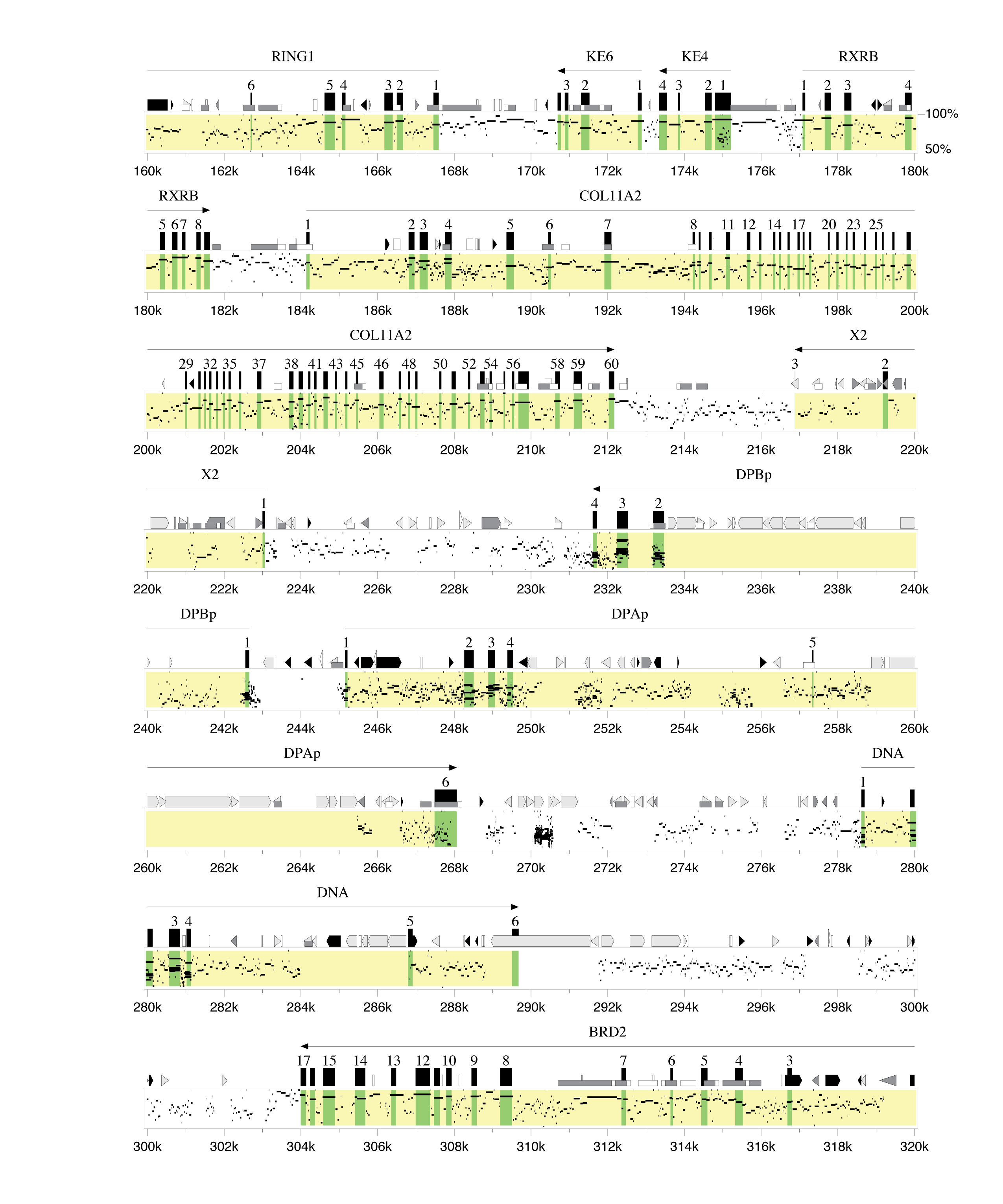

Supplement: Figure S1 — (3.98 MB BZ2) [file pone.0002674.s001.bz2 › Figure2A_2.png]

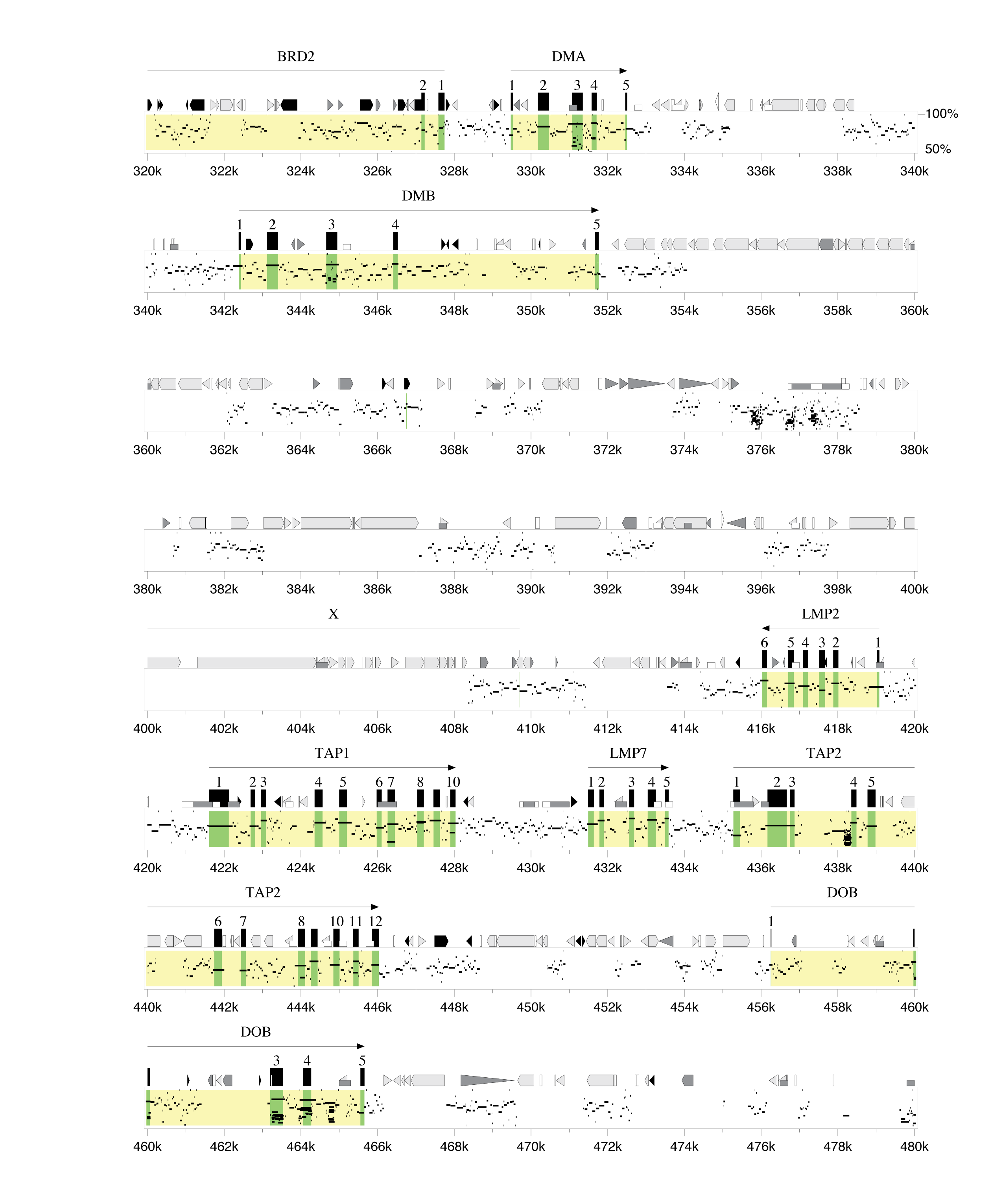

Supplement: Figure S1 — (3.98 MB BZ2) [file pone.0002674.s001.bz2 › Figure2A_3.png]

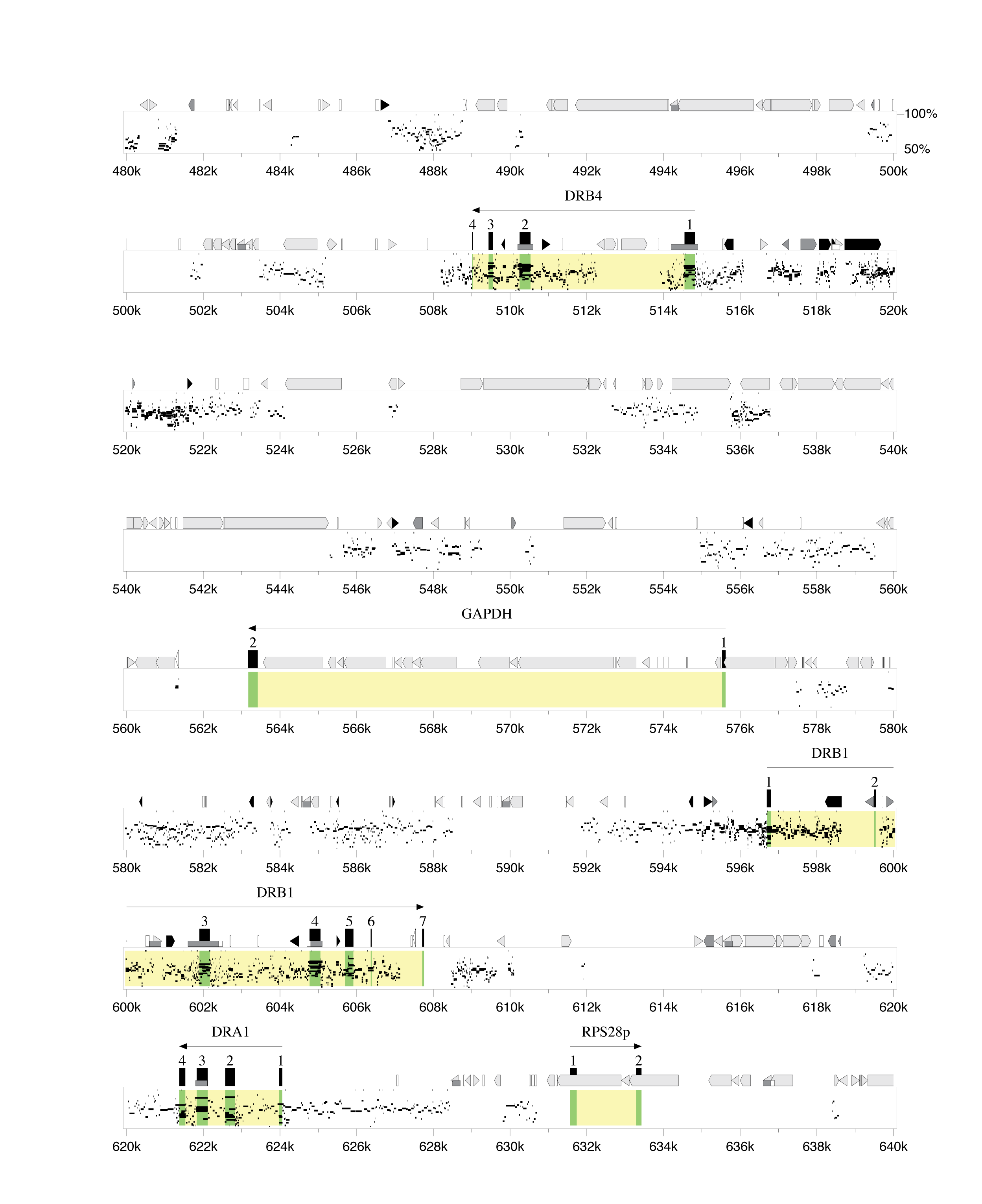

Supplement: Figure S1 — (3.98 MB BZ2) [file pone.0002674.s001.bz2 › Figure2A_4.png]

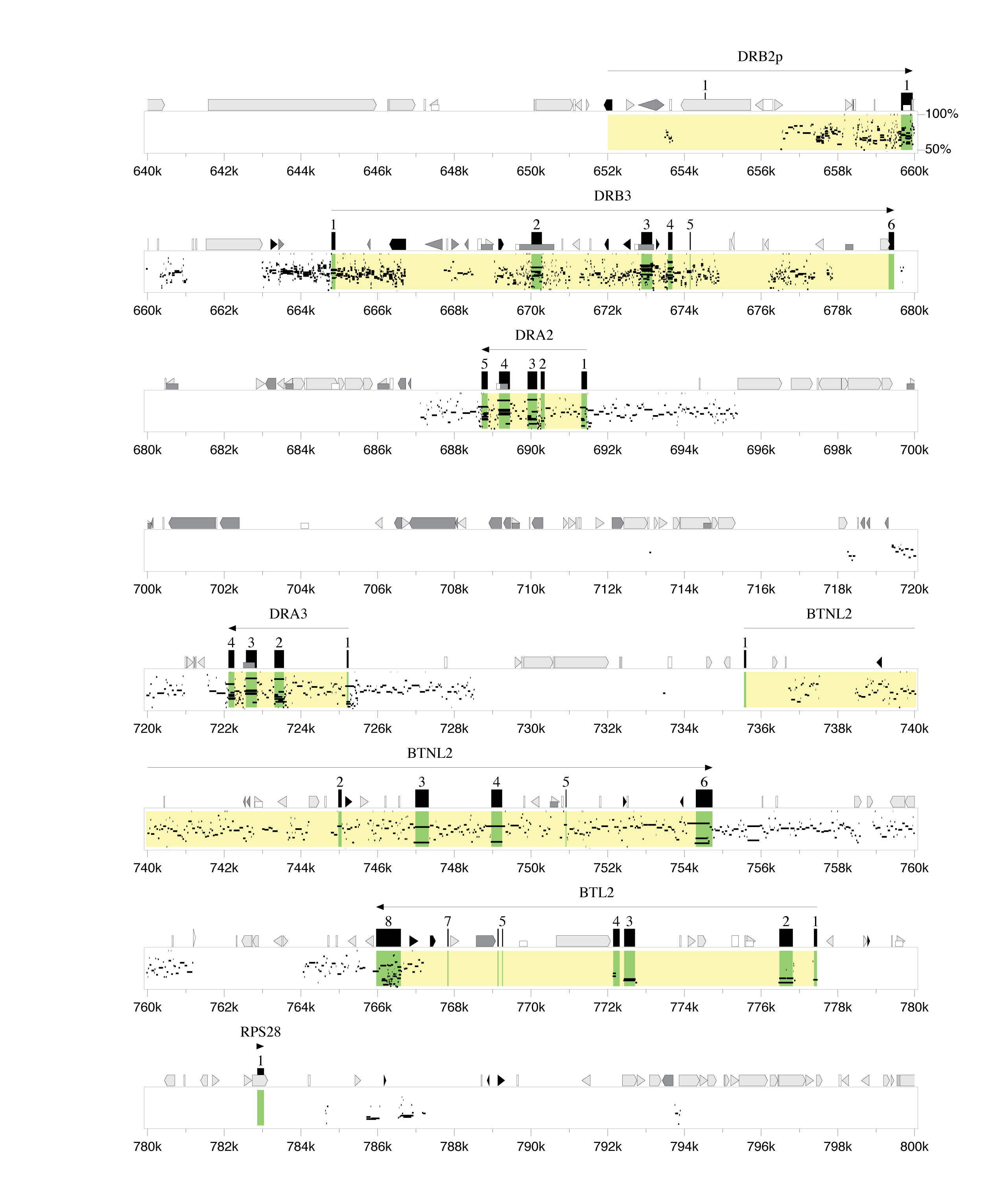

Supplement: Figure S1 — (3.98 MB BZ2) [file pone.0002674.s001.bz2 › Figure2A_5.png]

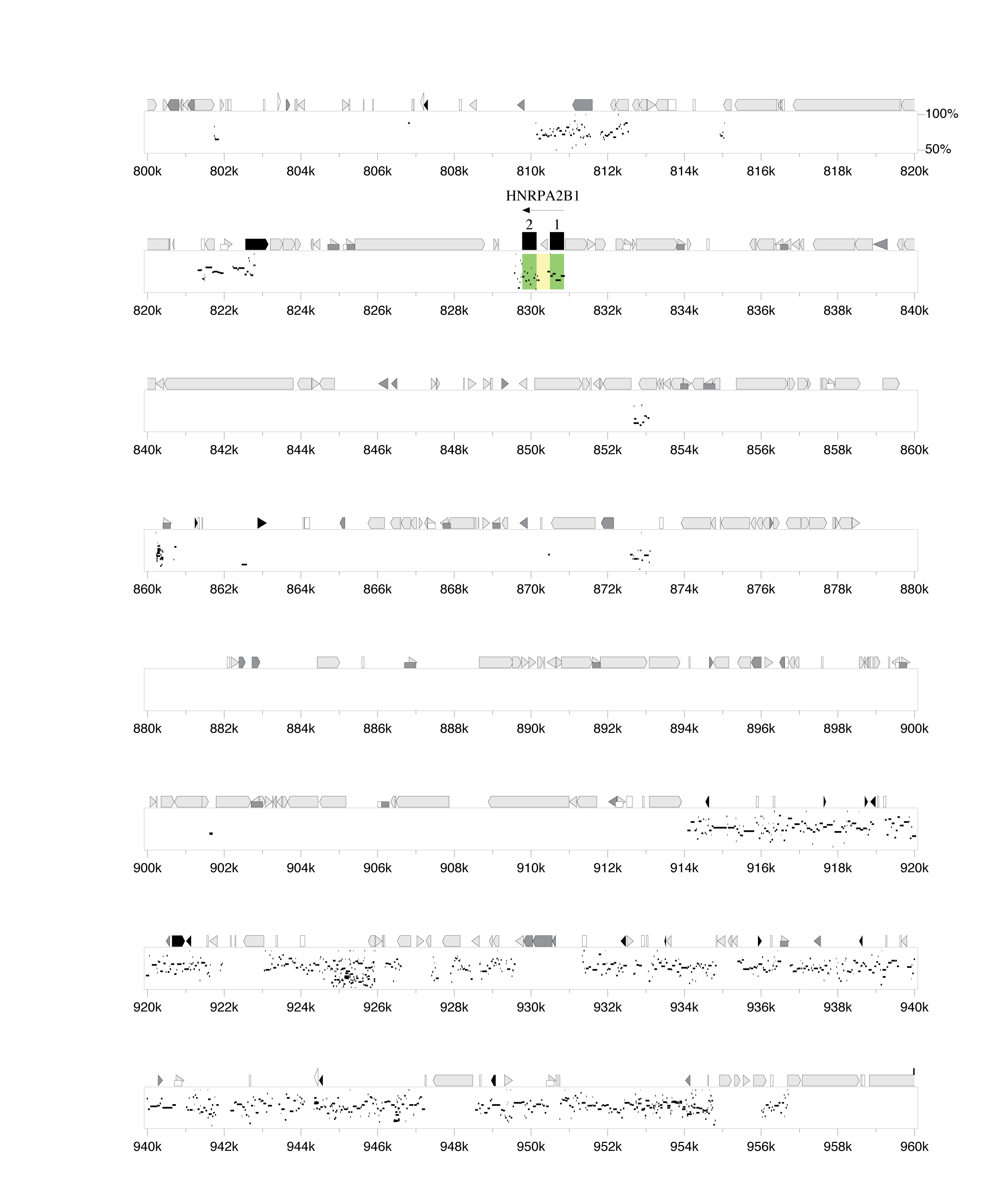

Supplement: Figure S1 — (3.98 MB BZ2) [file pone.0002674.s001.bz2 › Figure2A_6.png]

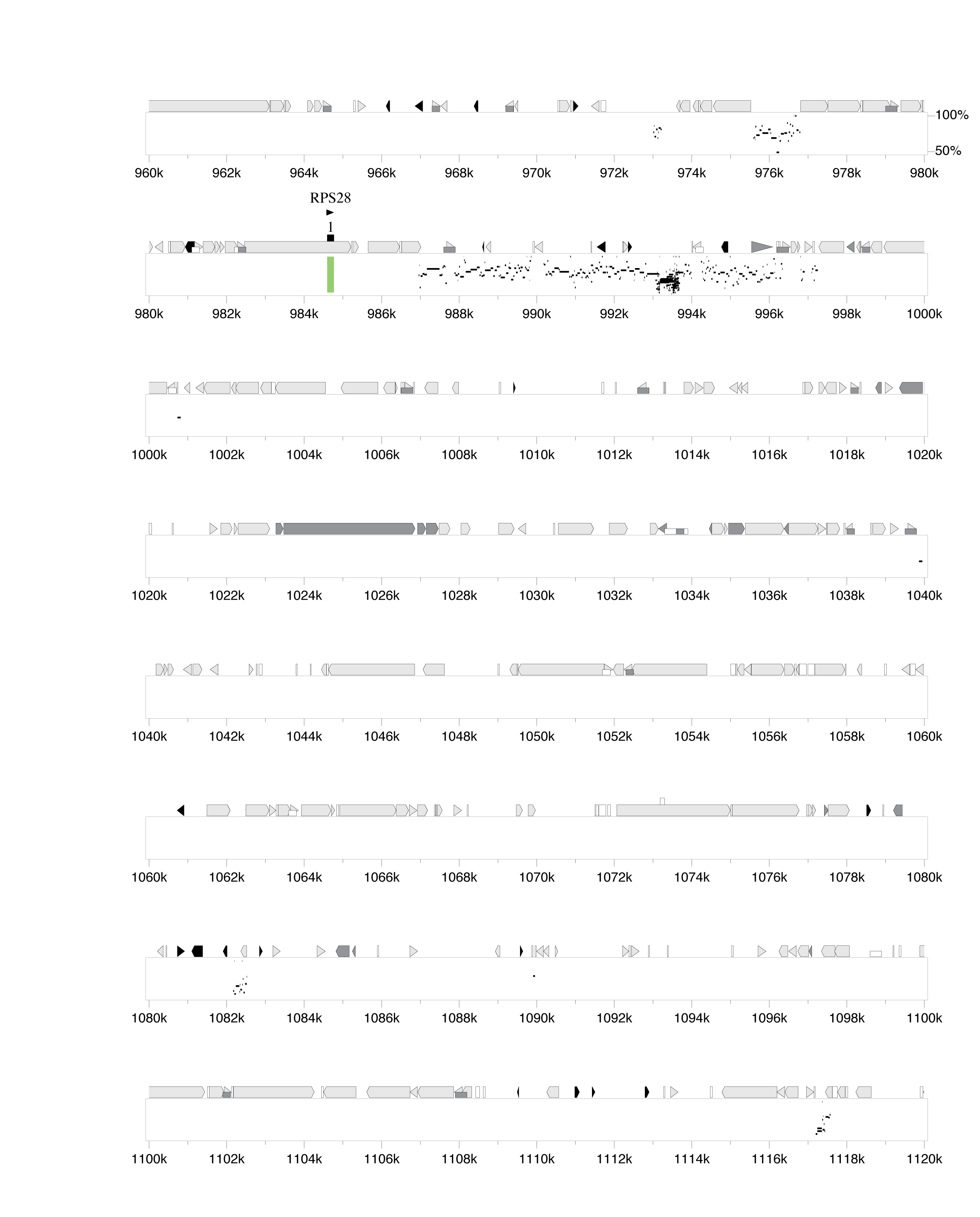

Supplement: Figure S1 — (3.98 MB BZ2) [file pone.0002674.s001.bz2 › Figure2A_7.png]

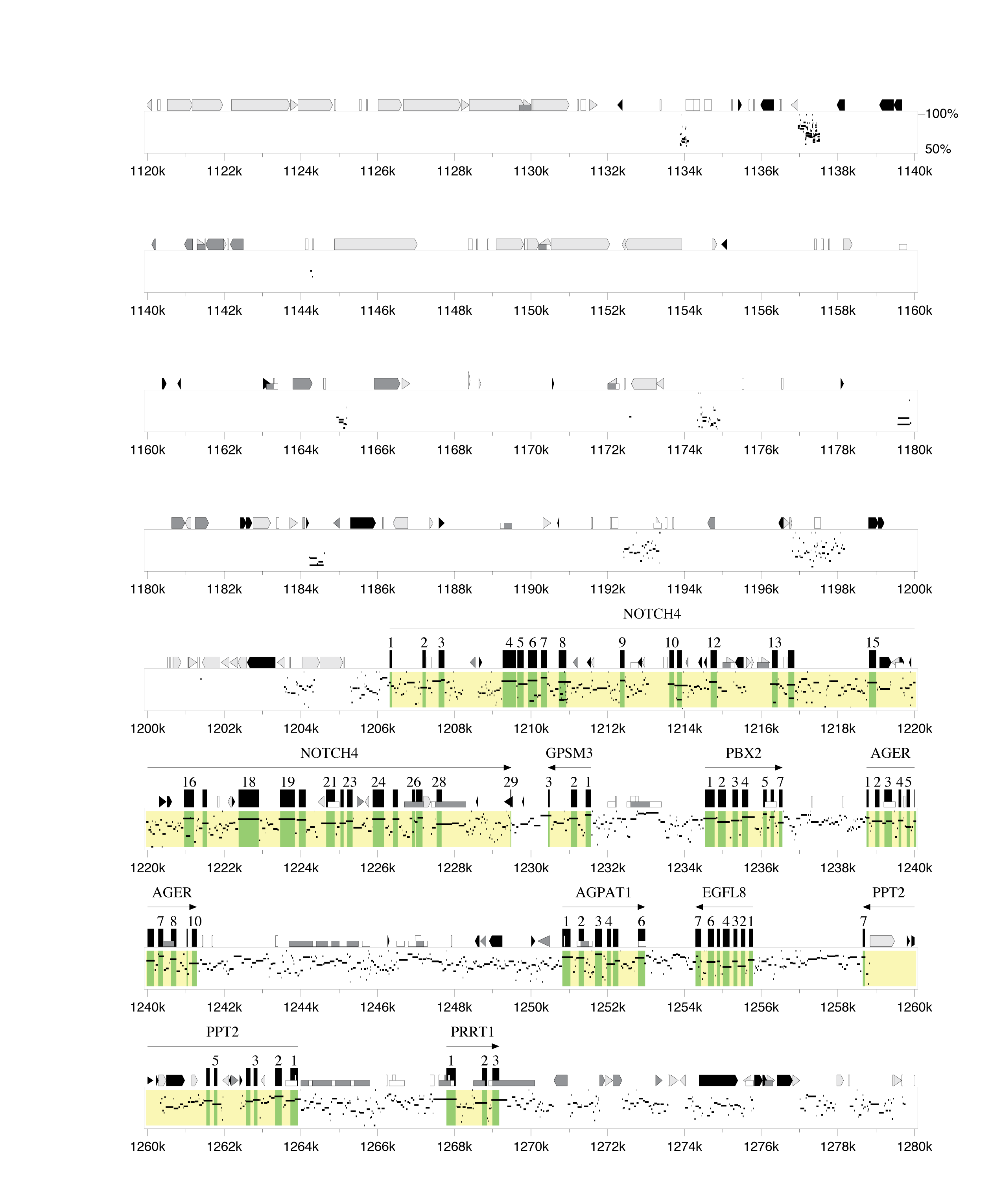

Supplement: Figure S1 — (3.98 MB BZ2) [file pone.0002674.s001.bz2 › Figure2A_8.png]

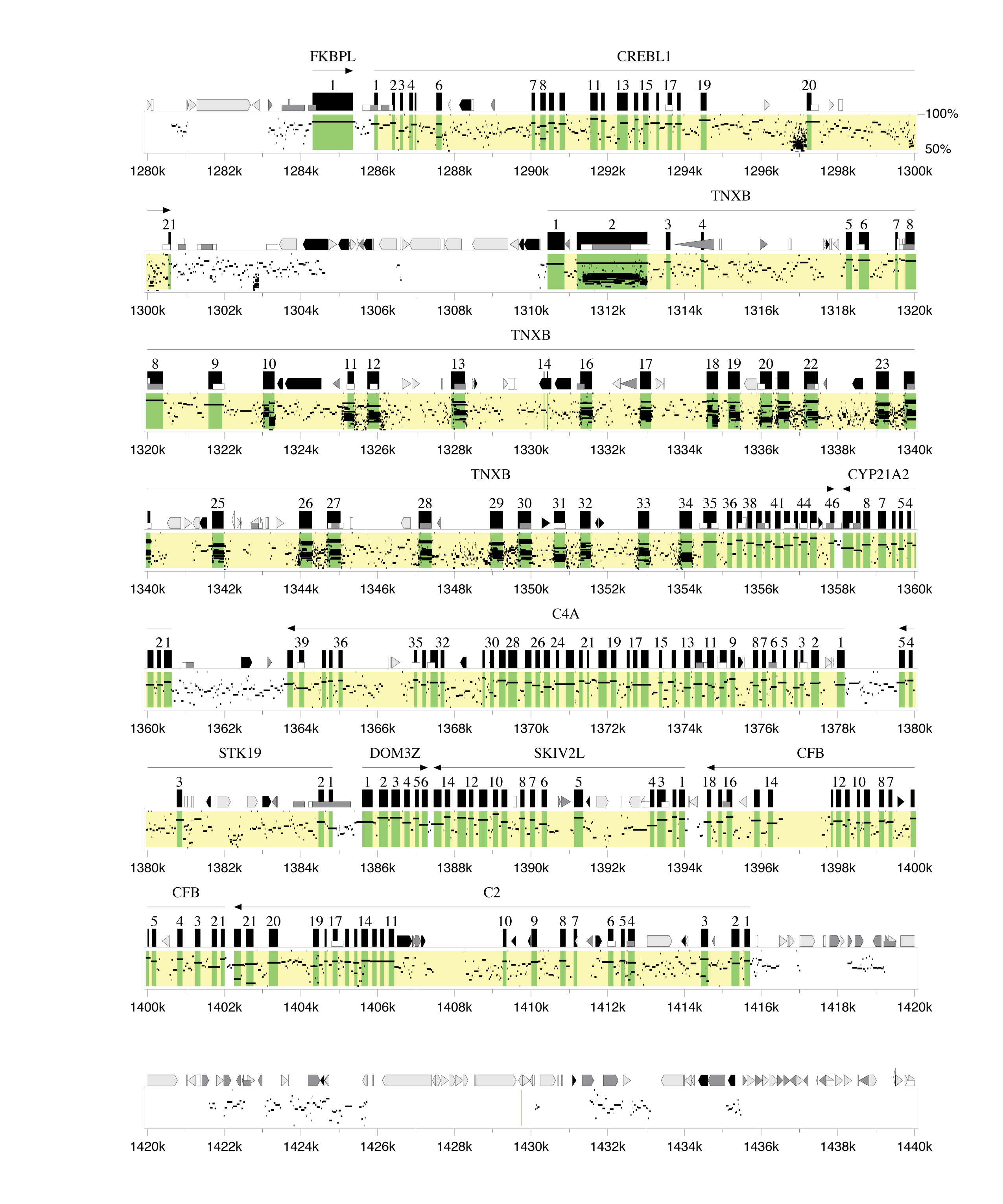

Supplement: Figure S1 — (3.98 MB BZ2) [file pone.0002674.s001.bz2 › Figure2A_9.png]

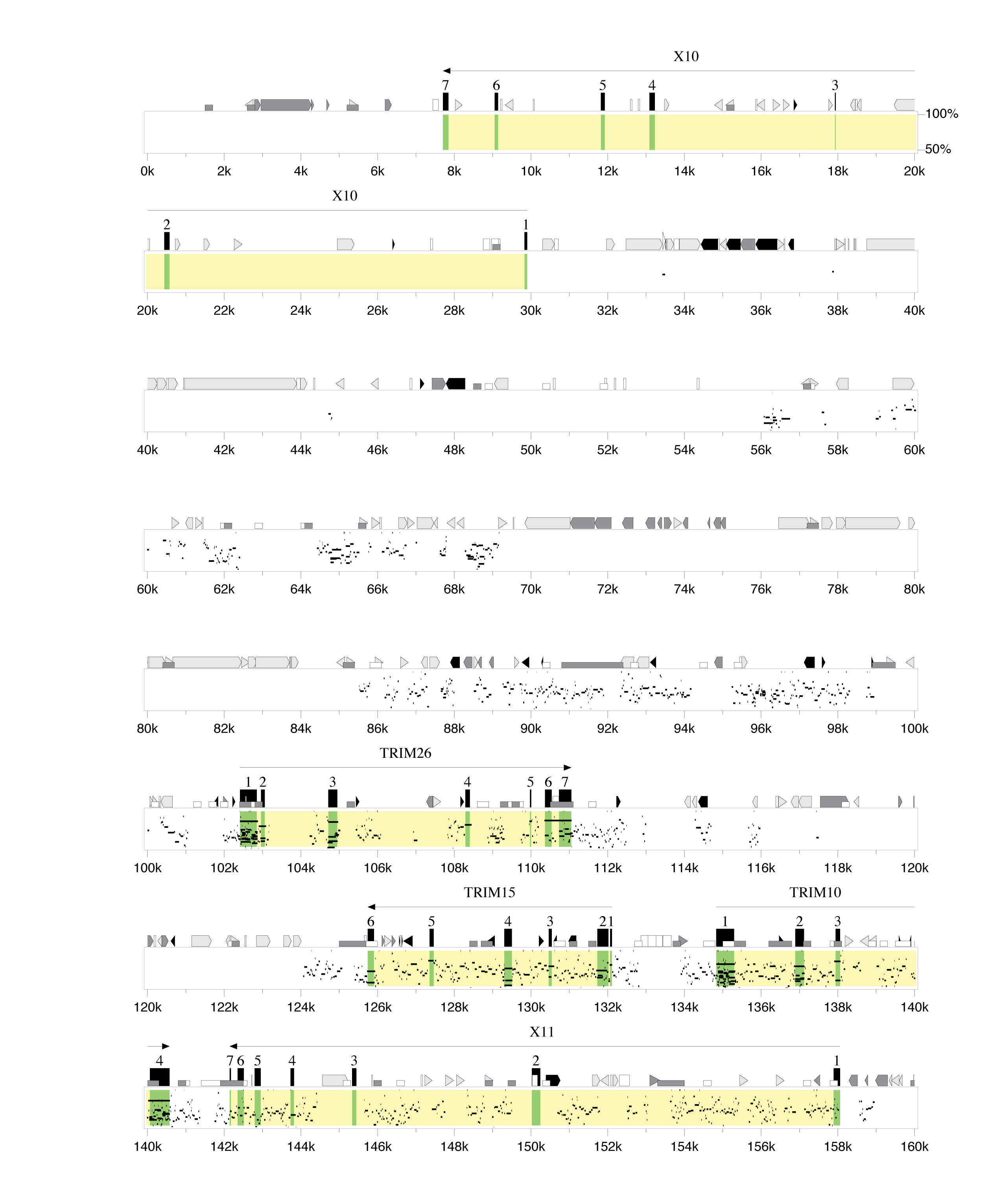

Supplement: Figure S1 — (3.98 MB BZ2) [file pone.0002674.s001.bz2 › Figure2B_1.png]

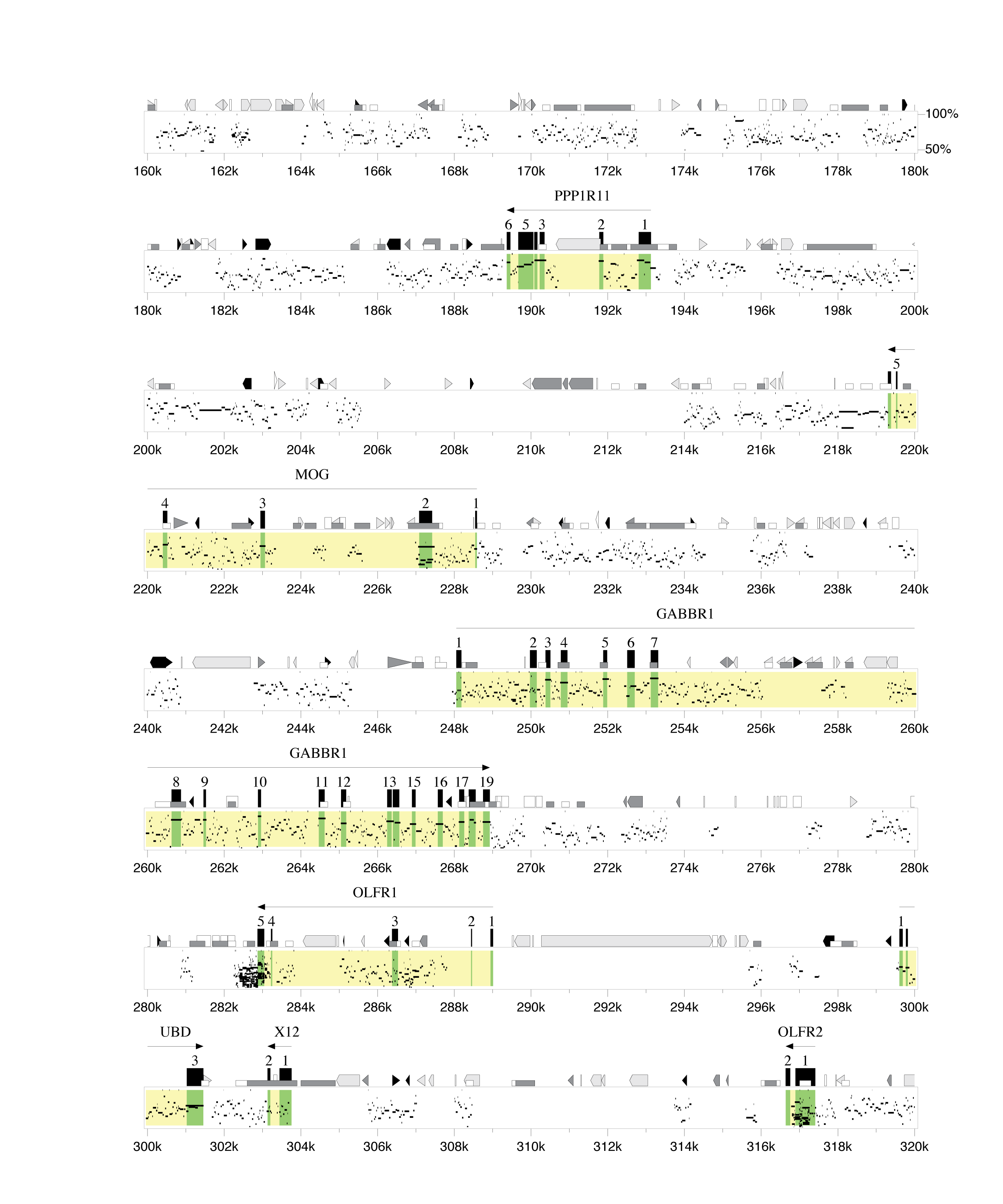

Supplement: Figure S1 — (3.98 MB BZ2) [file pone.0002674.s001.bz2 › Figure2B_2.png]

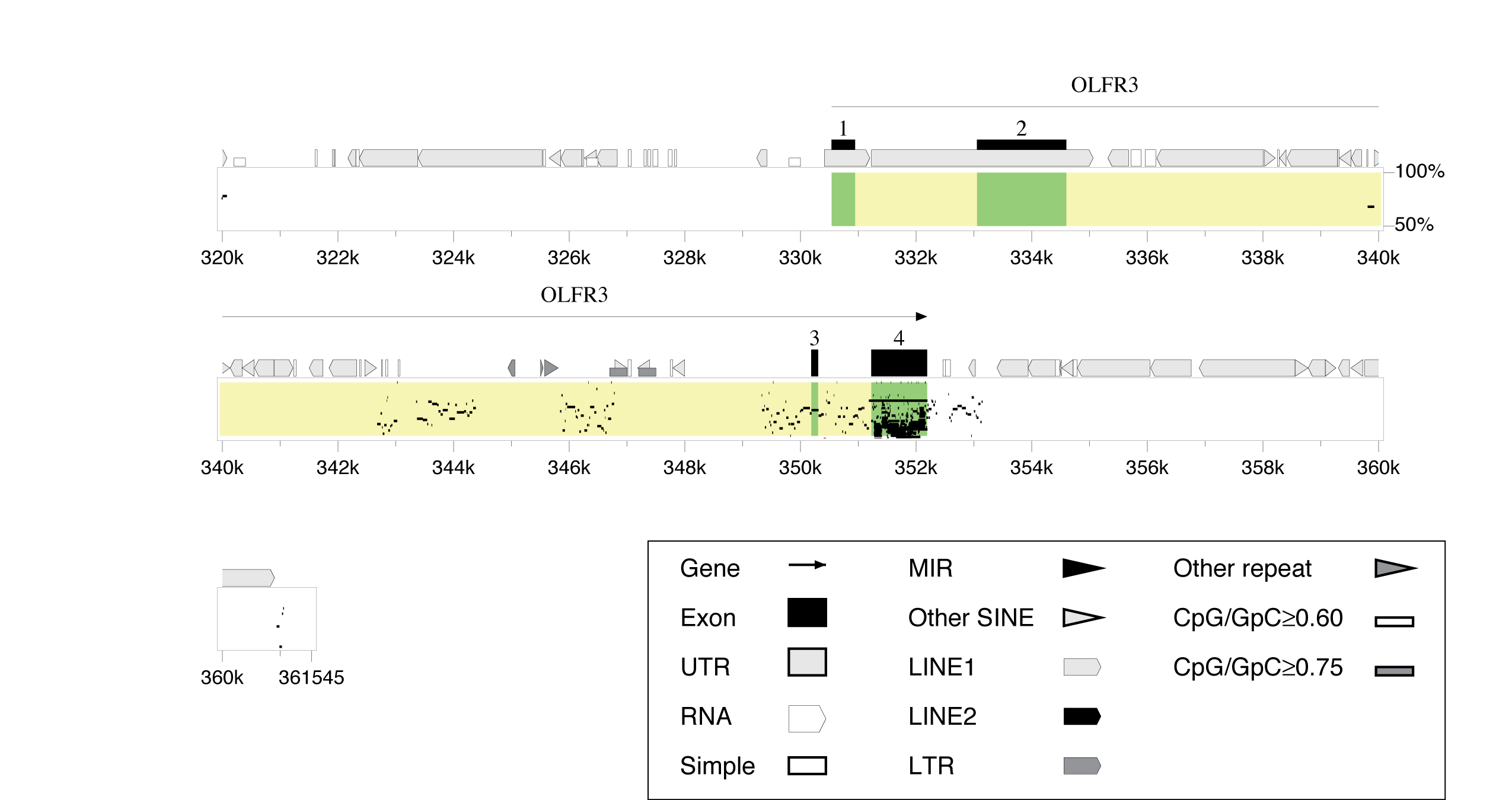

Supplement: Figure S1 — (3.98 MB BZ2) [file pone.0002674.s001.bz2 › Figure2B_3.png]
